# Structural basis of ubiquitin‐independent PP1 complex disassembly by p97

**DOI:** 10.15252/embj.2022113110

**Published:** 2023-06-02

**Authors:** Johannes van den Boom, Guendalina Marini, Hemmo Meyer, Helen R Saibil

**Affiliations:** ^1^ Molecular Biology I, Center of Medical Biotechnology, Faculty of Biology University of Duisburg‐Essen Essen Germany; ^2^ Centre for Structural Systems Biology Leibniz‐Institute of Virology and University Medical Center Hamburg‐Eppendorf (UKE) Hamburg Germany; ^3^ Biological Sciences, Institute of Structural and Molecular Biology Birkbeck University of London London UK

**Keywords:** AAA ATPase, cryo‐EM, protein phosphatase‐1, protein unfolding, Post-translational Modifications & Proteolysis, Structural Biology

## Abstract

The AAA+‐ATPase p97 (also called VCP or Cdc48) unfolds proteins and disassembles protein complexes in numerous cellular processes, but how substrate complexes are loaded onto p97 and disassembled is unclear. Here, we present cryo‐EM structures of p97 in the process of disassembling a protein phosphatase‐1 (PP1) complex by extracting an inhibitory subunit from PP1. We show that PP1 and its partners SDS22 and inhibitor‐3 (I3) are loaded tightly onto p97, surprisingly via a direct contact of SDS22 with the p97 N‐domain. Loading is assisted by the p37 adapter that bridges two adjacent p97 N‐domains underneath the substrate complex. A stretch of I3 is threaded into the central channel of the spiral‐shaped p97 hexamer, while other elements of I3 are still attached to PP1. Thus, our data show how p97 arranges a protein complex between the p97 N‐domain and central channel, suggesting a hold‐and‐extract mechanism for p97‐mediated disassembly.

## Graphical abstract

Cryo‐EM structures of p97/VCP‐p37 loaded with a regulatory PP1 complex illustrate how one PP1 partner, I3, is threaded through the ATPase's central channel, while another one, SDS22, remains tightly bound to the p97 N‐domain.

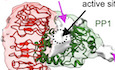

## Introduction

The AAA+ ATPase p97 governs diverse cellular signaling and stress response pathways including ER‐associated degradation, ribosomal quality control, DNA replication and damage repair, protein phosphatase‐1 (PP1) biogenesis, and autophagy that together ensure cell survival, proliferation, and homeostasis. In all these processes, p97 is believed to unfold substrate proteins often to dissociate clients from cellular structures or to disassemble protein complexes (Stach & Freemont, 2017; Ye *et al*, 2017; van den Boom & Meyer, 2018). p97 is considered a drug target in certain cancers and clinical trials are under way (Anderson *et al*, 2015; Skrott *et al*, 2017; Roux *et al*, 2021), whereas missense mutations in p97 cause a dominantly inherited multisystem proteinopathy‐1 (MSP‐1) featuring inclusion body myopathy, Paget's disease of bone, amyotrophic lateral sclerosis, frontotemporal dementia, and Parkinsonism (Kimonis *et al*, 2008).

Each subunit of the p97 hexamer contains two AAA+ ATPase domains, D1 and D2, that form two stacked hexameric rings enclosing a central channel, while the regulatory N‐domains are positioned at the periphery of the D1 ring (Zhang *et al*, 2000; DeLaBarre & Brunger, 2003; Banerjee *et al*, 2016). Biochemical reconstitution revealed that substrate proteins are inserted in the D1 pore of p97, threaded through the central channel and ejected from the D2 pore (Bodnar & Rapoport, 2017; Weith *et al*, 2018). Cryo‐electron microscopy (cryo‐EM) analysis showed that the active p97 hexamer is in a staircase configuration that allows the substrate threading mechanisms typical for AAA+ unfoldases (Cooney *et al*, 2019; Twomey *et al*, 2019; Pan *et al*, 2021; Xu *et al*, 2022). Aromatic residues in the pore loops engage in non‐sequence‐specific interactions with the substrate peptide backbone. Substrate threading occurs in a sequential hand‐over‐hand mechanism in which ATP hydrolysis in the subunit at the bottom of the p97 spiral results in a discontinuity as it triggers the detachment of this subunit from the spiral and induces its reattachment to the substrate peptide at the top. Thus, the peptide is pulled through the pore with the progression of nucleotide binding and hydrolysis around the ring.

For p97, substrate recruitment and engagement are mediated by at least two alternative types of substrate adapters that bind to the N‐domains of p97 (Buchberger *et al*, 2015; van den Boom & Meyer, 2018). Many p97 substrates are ubiquitylated and handled by the Ufd1‐Npl4 adaptor that binds the ubiquitin chain and inserts one ubiquitin into the D1 pore leading to threading of the attached substrate through the channel for substrate unfolding (Twomey *et al*, 2019). In contrast, ubiquitin‐independent substrate recruitment is mediated by adapters of the SEP‐domain family such as p37 (also called UBXN2B). Most prominently, p97‐p37 regulates the biogenesis of PP1 holoenzymes by disassembling a regulatory complex of PP1 catalytic subunit (called PP1 from here on) with its partners SDS22 (also called PPP1R7) and Inhibitor‐3 (I3, also called PPP1R11; Weith *et al*, 2018). Disassembly is achieved by threading I3 through the p97 channel which is speculated to strip I3 (and SDS22) off PP1 to allow PP1 holoenzyme formation with activating PP1 subunits (Weith *et al*, 2018). Intriguingly, I3 targeting involves an internal recognition site within I3 that is inserted as a loop into the p97 pore to initiate unfolding of I3 (van den Boom *et al*, 2021).

It is, however, unclear how protein complexes such as SPI (SDS22‐PP1‐I3) are loaded onto p97 for disassembly so that the direct substrate can be delivered into the pore whereas other subunits in this complex are spared. Moreover, no structural information exists on how ubiquitin‐independent targeting to p97 by the p37 adaptor is achieved. Therefore, the mechanism of PP1 activation in particular, and protein complex disassembly in general, is not understood.

Here, we have analyzed assemblies of p97 with its p37 adapter and the SPI substrate complex in the presence of ADP‐BeF_x_ by cryo‐EM, yielding a set of structures of p97‐p37 during the disassembly of the SPI substrate. Unexpected findings provide new insights into the spatial arrangement of a ubiquitin‐independent substrate and the adapter protein on p97 that facilitates complex disassembly.

## Results

### Human p97 in the act of translocating a specific substrate protein

**Figure 1:**
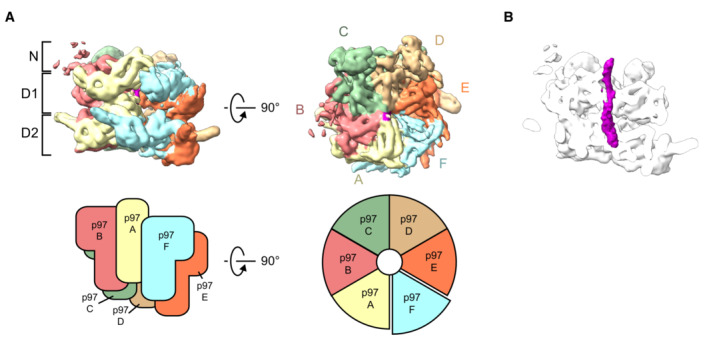
Structure of human p97 during I3 substrate translocation Side (left) and top (right) views of the cryo‐EM map of the p97 hexamer (0.00851 threshold). In the top view, subunit A, pale yellow, is followed by subunits B to F arranged in a clockwise staircase. Schematic views of the subunit assembly are shown in the lower panel.Side view section of p97 showing the I3 substrate density (purple) in the central channel. This map was determined before 3D classification into the different substrate complexes, and the flexible regions containing substrate were excluded by masking.

**Figure EV1:**
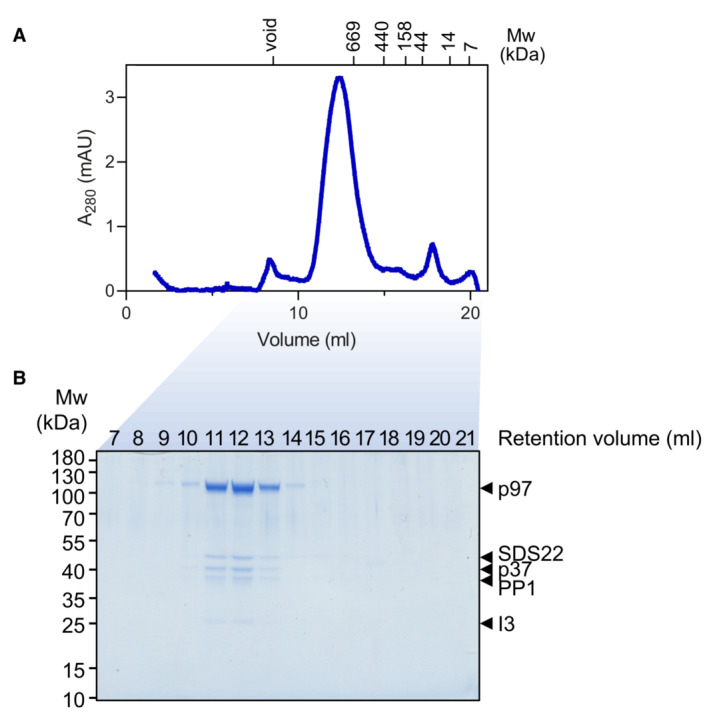
Purification of the p97‐p37‐SDS22‐PP1‐I3 complex Size exclusion chromatogram of equimolar concentrations of p97‐p37‐SDS22‐PP1‐I3 complex on a Superose 6 10/300 increase column. Molecular weights of reference standard are indicated.Coomassie gel of indicated size exclusion chromatography fractions from (a). Individual components of the complex are labeled.

**Figure EV2:**
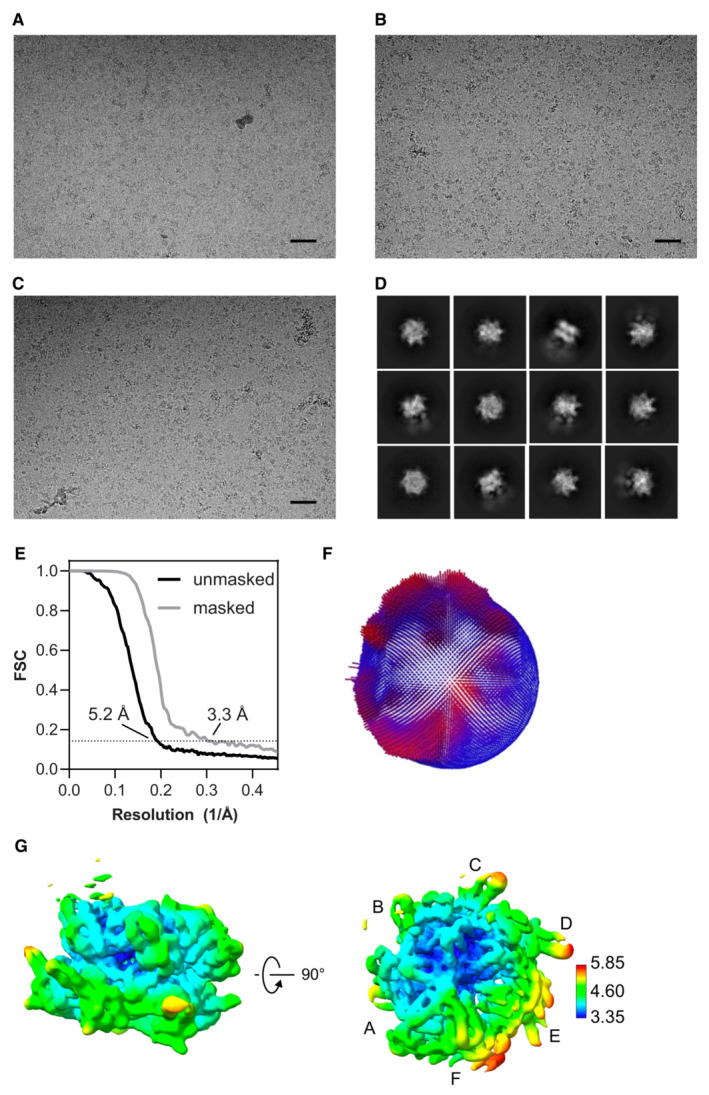
Cryo EM data and map validation for p97 A–CExample raw images from the particle dataset. Scale bar, 500 Å.DExample 2D class averages, showing some different particle orientations, with some weak substrate densities adjacent to the stronger p97 features. The box size is 352 × 352 Å.EGold‐standard Fourier shell correlation (GSFSC) calculated during refinement without and with mask. The resolutions were determined at FSC = 0.143 (dotted line).FPlot of particle orientations.GLocal resolution was calculated from the half‐map and colored according to the scale on the side. p97 subunits A–F labeled. For this map 866,937 particles were used.

**Figure EV3:**
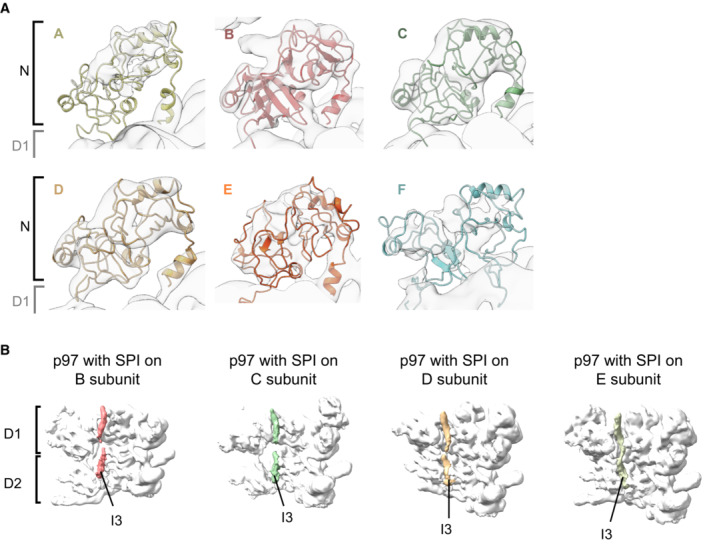
p97 N‐domains and density of threading substrate polypeptide Zoomed‐in view of p97 N‐terminal domains seen on the map with SPI bound to subunit B with the atomic models docked in. All six N‐domains of the p97 hexamer are clearly defined by the density to be in the “up” conformation.Views of the I3 substrate in the p97 pore of the maps with SPI bound on p97 subunits B, C, D, and E as indicated. Three protomers in the front of the p97 hexamer were removed for clarity.

To obtain structural data on how p97‐p37 disassembles the SPI complex, we added an excess of p97 and p37 to the SPI complex. Substrate engagement was induced by incubation in the presence of ADP and beryllium fluoride (ADP‐BeF_x_) and analyzed by cryo‐EM. In parallel, samples were analyzed by size exclusion chromatography and SDS–PAGE for quality control (Fig EV1A and B). Our structures show p97 loaded with the SPI complex at a defined position on the N‐domain in the act of translocating a segment of I3. The density map (Figs 1A and B, and EV2A–G) confirms a staircase configuration previously proposed for p97 and yeast Cdc48 (Cooney *et al*, 2019; Twomey *et al*, 2019; Pan *et al*, 2021; Xu *et al*, 2022) and conserved in other AAA+ proteins. Both D1 and D2 domains of p97 are in the staircase configuration with subunit A on top, followed by subunits B, C, and D in a clockwise manner to subunit E at the bottom (Fig 1A and B). Comparison with the model of Xu *et al* (2022; pdb 7mhs) shows an RMSD of 2.1 Å (Appendix Fig S1), confirming the high similarity of the p97 D1 and D2 domains (subunit F and the N‐domains were not included in the Xu *et al*, 2022 structure). Subunit F bridges the bottom subunit E with the top subunit A and has weaker electron density (Figs 1A and EV2G), likely reflecting its flexible position. All six N‐domains of p97 are in the “up” conformation, albeit the resolution for the A and F subunits is lower than for subunits B‐E (Fig EV3A). The central pore is filled in both ATPase rings with a density of an extended polypeptide strand (Fig 1B) that is engaged for threading via interactions to the D1 and D2 domains of five p97 subunits (A–E).

There is previous biochemical evidence that p97 initiates the translocation of I3 from a position close to the N terminus of the protein, taking a short peptide hairpin of I3 through the pore (van den Boom *et al*, 2021). Our map shows density corresponding to a single strand of I3 within the D1 ring pore, while the density inside the D2 ring pore is wider and compatible with a hairpin loop of I3 (Fig 1B). However, the resolution of the density for the mobile and disordered substrate is insufficient to reliably distinguish between single and double peptide strands.

### The SPI substrate complex is recruited to the N‐terminal domain of p97

**Figure 2:**
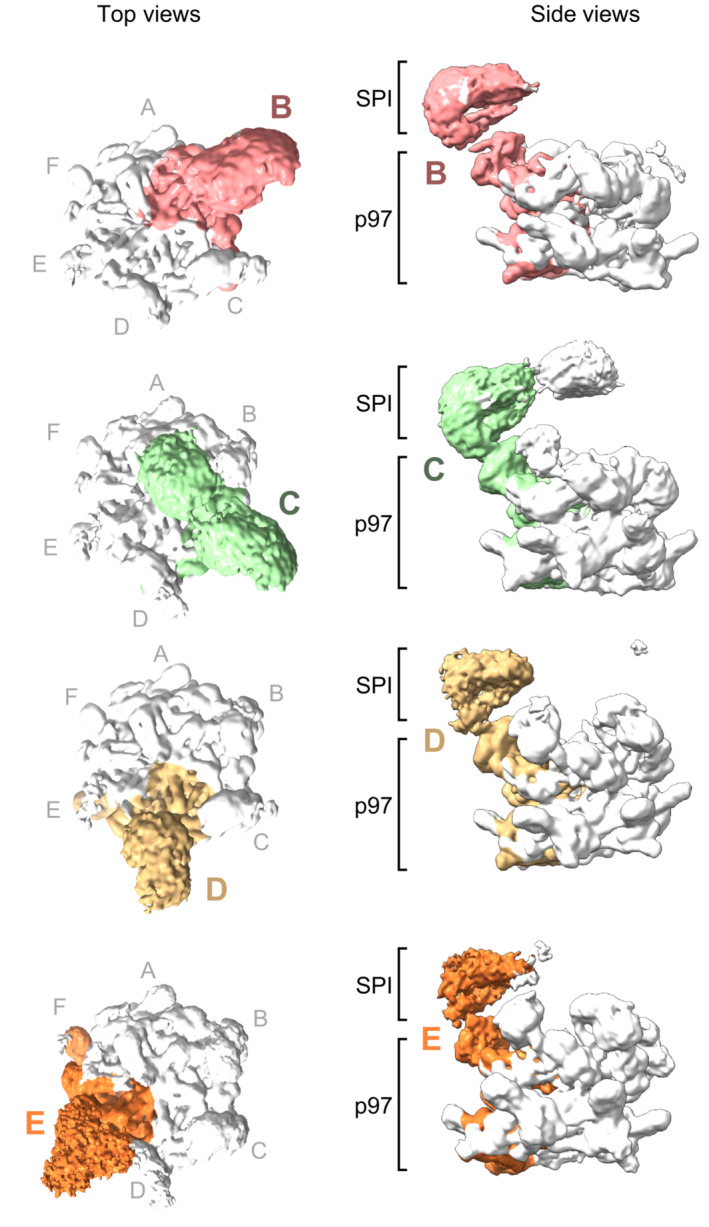
p97‐p37 loads the SDS22‐PP1‐I3 (SPI) substrate complex onto its N‐terminal domain Top and side views of cryo‐EM maps of four different structural classes showing density of one SPI complex above the N‐terminal domain in p97 subunits B, C, D, or E, respectively. An extra density above the channel was seen for the subunit C complex, which did not include enough images to group this feature into a separate class, as was done for the other subunit complexes. Subunits F and A showed weak density for SPI due to the flexibility of their interface. p97 subunits labeled starting from A as topmost subunit. Density thresholds: B, 0.00749; C, 0.00363; D, 0.0032; E, 0.00241.

**Figure 3:**
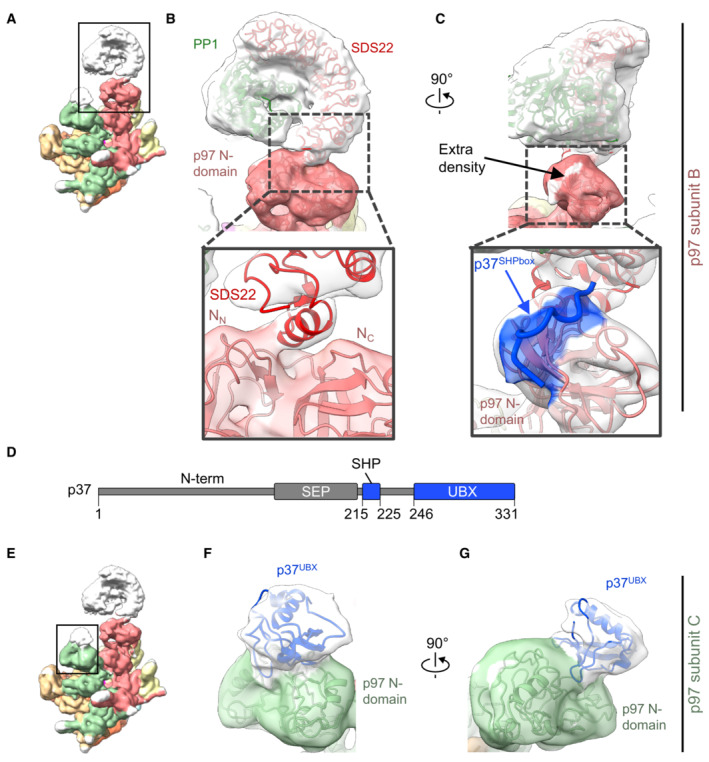
Position of SDS22 and p37 on the p97 N‐domains AShows the complex with the SPI substrate loaded on the B subunit of p97. Zoomed region is framed.BSDS22 interacts with the N‐domain of p97 (subunit B) via its C‐terminal helix and binds PP1 on its entire concave face. Zoom‐in view shows the interface between SDS22 and the p97 N‐domain. SDS22 binds with its C‐terminal helix in the groove between the N‐ and C‐terminal lobes (N_N_ and N_C_) of the p97 N‐domain.CRotated view showing the extra density (white) at the position of the conserved SHP box binding site. Zoom‐in view shows the SHP box peptide (blue) based on PDB 5B6C modeled into the density.DDomain composition of the p37 adapter. N‐term: unstructured N terminus; SEP: SEP (Shp1, eyes closed, p47) domain; SHP: SHP box for p97 binding; UBX: ubiquitin regulatory X (UBX) domain for p97 binding. Parts resolved in the structure are depicted in blue. Residue numbers of structural elements are indicated.EShows the same map as in (a). Zoomed region is framed.F, GThe N‐domain of the p97 C subunit (green) adjacent to the SPI‐bound B subunit binds the UBX domain of p37 (blue). Data information: Density threshold of the map is 0.0024.

**Figure EV4:**
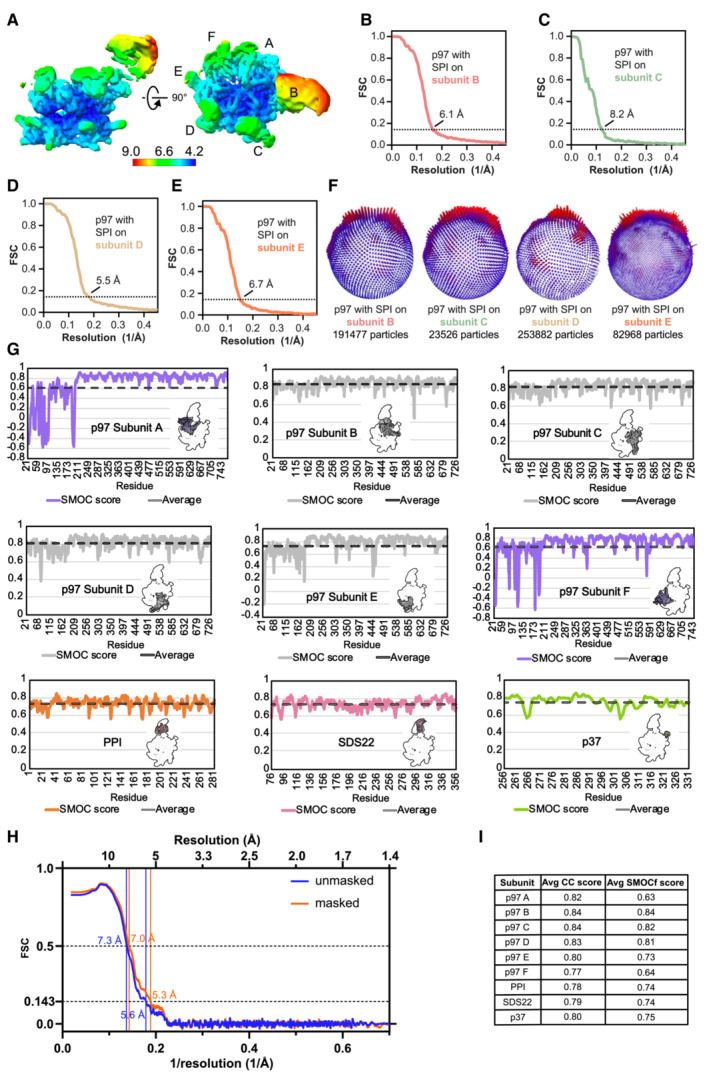
Validation information for p97 complex maps and model fitting ALocal resolution of the p97 map with SPI bound on subunit B colored according to the scale below. The resolution scale mainly serves to show the relative resolutions of different parts of the structure. The absolute values for the mobile N and substrate domains appear too optimistic.B–EGold‐standard Fourier shell correlation (FSC) plot for the p97 map with SPI bound on the B subunit (B), C subunit (C), D subunit (D), and E subunit (E). Resolutions were determined at FSC = 0.143 (dotted line).FParticle orientation distribution and particle numbers indicated below for the p97 map with SPI bound on the B, C, D, or E subunit.GSMOCf score plots are shown per residue for every subunit. Dashed lines indicate average values.HPlot of the map‐to‐model FSC with and without mask showing similar FSC curves and resolutions. Resolutions were determined at FSC = 0.143 and 0.5 (dashed lines), as indicated.ISummary of the average CC and SMOCf scores for each subunit. All FSCs were calculated without masking.

Our image dataset was sorted into classes yielding maps representing different populations of SPI‐loaded p97 complexes, with SPI bound to N‐terminal domains of p97 subunits at different positions in the hexameric staircase. We obtained four individual maps, in which the SPI complex is positioned on the second, third, fourth, and fifth p97 subunit (B, C, D, and E subunit), respectively (Figs 2 and EV3B and EV4A‐I). The SPI densities occupy a consistent position above the different p97 N‐terminal domains with weaker densities extending slightly toward the channel entrance near the central axis of p97. This suggests that the SPI substrate complex remains bound to the same p97 subunit which then, together with SPI, proceeds through the different positions in the staircase configuration (A–F) during the translocation cycle. All four maps show a density of the I3 substrate in the p97 pore (Fig EV3B). Subunit B shows the best‐defined SPI density, whereas the density becomes more disordered for subunits C, D and E (Fig 2). We therefore focused our analysis on the structure with SPI bound on the B subunit of p97 (Fig 3A–G).

### SDS22 docks in the p97 N‐domain groove while p37 spans two adjacent N‐domains

**Figure 4:**
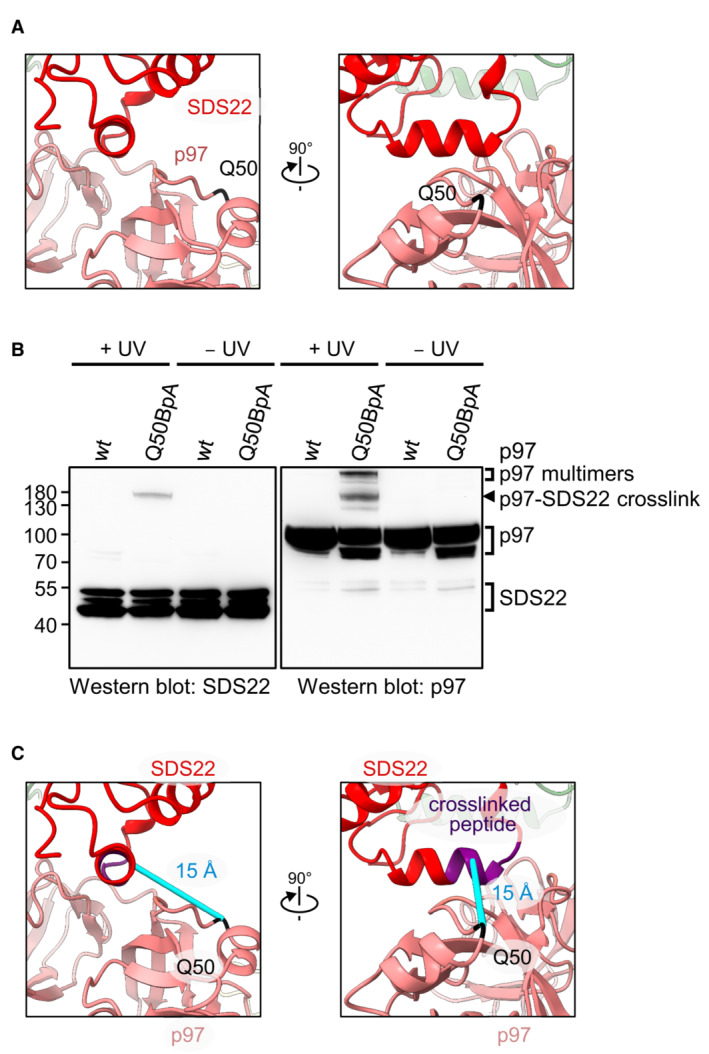
C‐terminal helix in SDS22 interacts directly with p97 Position of the genetically encoded crosslinker at the interaction site between SDS22 and the p97 N‐domain groove. Q50 in the p97 N‐domain was replaced by a genetically encoded BpA crosslinker.Western blot analysis of cross‐links between p97 and SDS22. A BpA crosslinker was genetically introduced at residue Q50 in the p97 N‐domain near the SDS22 interface. Wild‐type p97 or p97‐Q50BpA was incubated with p37, SPI, and ATP before cross‐linking by UV irradiation or mock treatment as indicated. p97‐SDS22 cross‐link, p97 interprotomer cross‐links (p97 multimers), as well as non‐cross‐linked p97 and SDS22 are labeled.The cross‐link between p97‐Q50BpA and SDS22 identified by mass spectrometry was mapped onto the cryo‐EM structure. The distance between the cross‐linked region in SDS22 (purple) and the BpA crosslinker (black) in the p97 B subunit is about 15 Å (cyan).

**Figure EV5:**
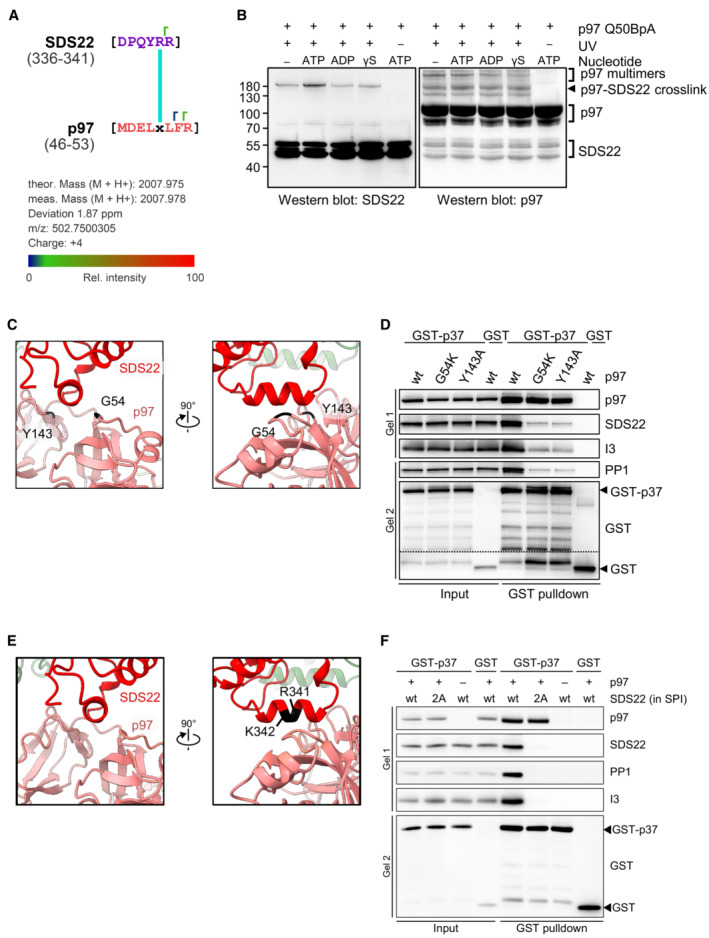
Biochemical analysis of the p97‐SDS22 interface p97‐Q50BpA cross‐link sample from Fig 4B was analyzed by mass spectrometry. One peptide with an FDR <5% was detected. The BpA at amino acid position 50 (× in the sequence) in p97 is cross‐linked to a peptide of SDS22 covering residues 336–341. Identified y ions are indicated (┌).Western blot analysis of cross‐links between p97 with BpA crosslinker at residue Q50 and SDS22 in the presence of different nucleotides (2 mM) as indicated. p97‐Q50BpA was incubated with p37, SPI and different nucleotides before cross‐linking by UV irradiation or mock treatment as indicated. p97‐SDS22 cross‐link, p97 interprotomer cross‐links (p97 multimers), as well as non‐cross‐linked p97 and SDS22 are labeled. γS denotes ATPγS.Views of the interaction site between SDS22 and the p97 N‐domain groove. Mutated residues in p97 chosen for proximity to the SDS22‐p97 contact site are labeled. Both G54 and Y143 are at 5.1 Å distance (Cα‐Cα distance) from the SDS22 helix.GST‐pulldown binding assays using purified GST or GST‐p37, SPI, and p97 variants with the chosen mutations as indicated were carried out in the presence of ATP. Western blot with indicated antibodies. Note that the mutations largely reduce SPI but not p37 binding.Views of the interaction site between SDS22 and the p97 N‐domain groove. Mutated residues in SDS22 chosen for proximity to the SDS22‐p97 contact site are labeled.GST‐pulldown binding assays using purified GST or GST‐p37, without or with p97 and with SPI or SPI variant with the chosen mutations (R341A + K342A, labeled 2A) as indicated were carried out in the presence of ATP. Western blot with indicated antibodies. Note that the mutations abolish SPI binding to p97‐p37.

The SPI complex on subunit B is seen very clearly as an arc‐shaped density enclosing a globular density (Fig 3A–C), corresponding well with the available X‐ray crystal structures of the PP1‐SDS22 complex, in which the leucine‐rich repeats (LRRs) of SDS22 form an arc around PP1 (Choy *et al*, 2019). It is generally assumed that substrates are recruited to the p97 N‐domain indirectly through substrate adapters (Buchberger *et al*, 2015; Stach & Freemont, 2017). Surprisingly, our structure shows a direct interaction of SDS22 with the N‐domain of p97 (Fig 3B). Closer analysis of this interface revealed docking of a short helix (aa 337–346), located C‐terminally of the LRR array in SDS22, to the groove between the N‐terminal double Ψ‐barrel (N_N_) and C‐terminal four‐stranded β‐barrel (N_C_) of the p97 N‐domain (Fig 3B, zoomed‐in view). This is in line with complementary charge distribution of the positive SDS22 helix and the negative p97 groove. We confirmed this interaction with the genetically encoded photo‐crosslinker p‐benzoyl phenylalanine (BpA) at position Q50 in the p97 N‐domain groove that cross‐linked to SDS22 and was mapped to the C‐terminal helix of SDS22 (Figs 4A–C and EV5A). Of note, the cross‐link was equal or even stronger in the presence of ATP compared with ADP, ATPγS or in the absence of nucleotide, suggesting that the interaction was stable even when p97 was active and pulling force was applied to the PP1 complex (Fig EV5B). We further validated this interaction with point mutations first in the p97 N‐domain groove (G54K or Y143A) and, conversely, in the SDS22 helix (R341A and K342A) that both largely reduced SPI binding, but not p37 binding, to p97 (Fig EV5C–F).

Substrate adaptors such as p37 and its paralogues, as well as Ufd1‐Npl4, interact through two different anchor points with the p97 N‐domain, a linear SHP box and a UBX domain (Fig 3D) (Bruderer *et al*, 2004). As previous structures only show these interactions individually (Dreveny *et al*, 2004; Hanzelmann & Schindelin, 2016; Le *et al*, 2016; Lim *et al*, 2016; Li *et al*, 2017; Cooney *et al*, 2019; Xu *et al*, 2022), it has remained controversial whether the SHP box and UBX domain dock to the same or different N‐domains within the p97 hexamer. In our structure, there is extra density on the p97 N‐domain at the SHP box binding site underneath the PP1 complex, which well accommodates the p37 SHP box segment (Fig 3C; Appendix Fig S2; Le *et al*, 2016). Of note, the UBX domain of the adapter cannot bind the same N‐domain as its interaction site in the N‐domain groove is occupied by SDS22. Instead, we see a UBX domain on the adjacent subunit (Fig 3E–G; Appendix Fig S2) with its N terminus facing toward the SHP box peptide. The distance between the SHP box and UBX domain on the adjacent N‐domain is 50 Å, which can easily be bridged by the 20 residues in the linker between SHP box and UBX domain. Therefore, our structural results point to an extended structure for the p37 adaptor, in which the SHP box peptide and UBX domain bind to successive p97 subunits. This configuration locates the linker underneath PP1, which is consistent with our previous finding that the linker is critical for interaction with PP1 and for recruitment of the SPI complex (Kracht *et al*, 2020). In support of this proposal, previous biochemical evidence showed that the active complex with the SPI substrate requires only one copy of p37 (Kracht *et al*, 2020). Also, Cooney *et al* (2019) only observed one UBX domain of the p37 orthologue Shp1.

### Assignment of additional I3 densities on PP1

**Figure 5:**
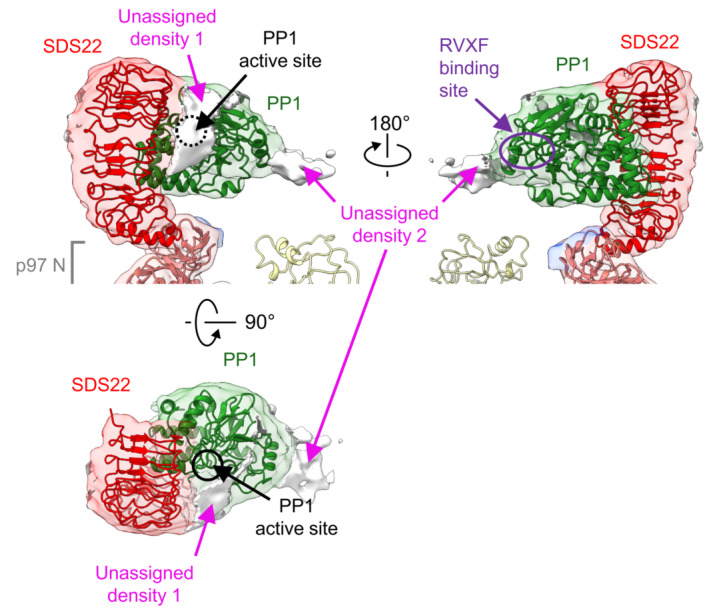
Structure of the SPI complex with p97 Side (upper panels) and top (lower panel) views the SPI complex of subunit B (threshold 0.0034) with fitted crystal structures of PP1 and SDS22 (PDB: 6obn). Additional density at the bottom of the upper panels is part of the N‐domain. There are two regions of unassigned density between PP1 and SDS22. Density 1 is next to the active site of PP1 (black dashed circle). Density 2 is in the vicinity of the binding site for the RVXF motif in I3 (purple dashed circle). The unassigned densities likely represent parts of I3.

**Figure 6:**
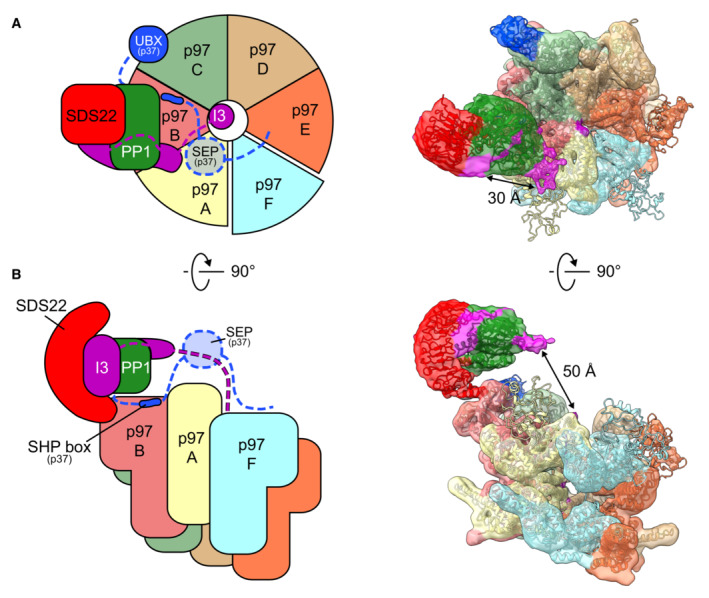
Schematic of proposed p97 processing of the SPI complex A, BTop view of p97‐p37 during disassembly of the SPI complex as cartoon (left panel) and density map with models (right panel, threshold 0.0034) in top (A) and side view (B). The p97 hexamer (subunits A–F) is in staircase conformation with subunit A on top and E on the bottom. The p37 adapter positions the SPI on the p97 B subunit of the p97 hexamer (subunits A‐F) with the p37 SHP box bound to the B subunit and the p37 UBX domain bound to the p97 N‐domain of the adjacent C subunit. The SPI complex is bound on the p97 hexamer via a direct interaction of the SDS22 C‐terminal helix with the p97 N‐domain of the B subunit. The concave side of SDS22 faces toward the p97 pore and is occupied by PP1. The unfolded polypeptide in the central channel of the p97 hexamer is likely the N‐terminal part of Inhibitor‐3 (I3) and interacts with the p97 subunits A–E, while the F subunit is disengaged. The RVXF motif of I3 is attributed to the electron density at the RVXF binding site of PP1, located 50 Å above the p97 pore, and possibly guided to the pore by the p37 SEP domain. The electron density covering the active site of PP1 likely reflects the inhibitory region in the C‐terminal part of I3.

The SPI density above the N‐domain of p97 contains two regions of additional density that are not filled by PP1 or SDS22 (Fig 5) suggesting they are occupied by I3. This is consistent with two binding sites of I3 to PP1 identified through interaction studies (Zhang *et al*, 2008) and cross‐linking (van den Boom *et al*, 2021). One unassigned density is adjacent to the PP1 active site (Fig 5 density 1). Binding of this region by a C‐terminal part of I3 (aa 65–77) is critical for the inhibitory activity of I3 for PP1 (Zhang *et al*, 2008). The other is on the opposite side of PP1, in the vicinity of the binding site in PP1 for the RVXF motif of I3 (aa 40–43 in I3), which is essential for I3‐PP1 interaction (Fig 5 density 2) (Zhang *et al*, 2008; van den Boom *et al*, 2021). The distance between these two sites is approximately 30 Å, which is compatible with the 21 residues between the two regions in I3 (Zhang *et al*, 2008). This suggests that a region near the RVXF motif and the inhibitory region of I3 account for the extra densities, while other parts of I3 which are not interacting with PP1 are likely to be disordered and therefore not visible in the density. The distance between the density at the tip of the SPI complex corresponding to the RVXF motif in I3 and the p97 pore that accommodates the proposed inserted N‐terminal section of I3 is approximately 50 Å (Fig 6A and B). This could be compatible with the roughly 40 amino acids N‐terminal of the RVXF motif that is first targeted for pore insertion (van den Boom *et al*, 2021).

## Discussion

Our structure of p97 with a loaded substrate complex on the N‐domain reveals unanticipated new principles of how a substrate complex is loaded onto p97 with the help of the adapter leading to the dissociation of one subunit while sparing the other components. The most prominent feature of our structure is the precise docking of the SPI substrate complex on the p97 N‐domain. This is suggested by the well‐defined structure of SDS22‐PP1 and its interaction interface with the N‐domain. Surprisingly, the interface is not solely conferred by the adapter as generally assumed. Instead, SDS22 appears to make direct contact with the groove between the two N‐domain lobes of the p97 N‐domain, which excludes interaction of the p37 UBX domain to the same N‐domain. In fact, our structure shows the p37 SHP box underneath the SPI complex and the UBX domain on the adjacent N‐domain. The distance can be readily bridged by the linker between SHP box and UBX domain, which however is not visible in our structure. The proposed position of the bridging linker is also consistent with previous data on a cross‐link between the linker and PP1 (Kracht *et al*, 2020). Thus, our new structural information together with previous biochemical data (Weith *et al*, 2018; Kracht *et al*, 2020) suggests that multivalent interactions of adapter, the p97 N‐domain and subunits of the SPI complex tightly dock and hold SDS22‐PP1 on the p97 N‐domain. The observed substrate‐loaded p97 complex likely represents the configuration for peptide threading initiation, which could rearrange during threading. However, the fact that the cross‐link sensor continues to detect the SDS22 interaction with p97‐N in the presence of hydrolysable ATP (Fig EV5B) speaks for a stable positioning of the SPI complex even during threading and thus suggests a hold‐and‐extract mechanism for the disassembly of the substrate complex.

The unassigned densities not filled by SDS22 or PP1 on the SPI complex correspond well with biochemically established I3 binding sites covering the catalytic site of PP1 and the RVXF interaction site in PP1 (Zhang *et al*, 2008; van den Boom *et al*, 2021), although we cannot exclude that the disordered N terminus of SDS22 also contributes to this density. Of note, the RVXF site points toward the D1 pore of p97 that harbors an inserted peptide apparently derived from I3. We previously demonstrated biochemically that a sequence element in I3 N‐terminal to the RVXF motif is recognized and engaged in the pore (van den Boom *et al*, 2021). Given that the distance between these densities is compatible with the 40‐residue length of that sequence, we speculate that the I3 elements on PP1 and in the channel are connected, and that our structure represents I3 in the process of being extracted from PP1 (Model Fig 6A and B; Movie EV1). The ATPase rings in our structure are in a spiral conformation primed to thread the peptide through the channel by the established hand‐over‐hand mechanism (Cooney *et al*, 2019; Twomey *et al*, 2019; Pan *et al*, 2021; Xu *et al*, 2022). The peptide which connects densities of I3 on PP1 and in the channel is likely flexible and therefore not visible in the density. Of note, previous cross‐link experiments suggested that the p37 adapter also binds I3 and that this is mediated by its SEP domain (Weith *et al*, 2018). As the SEP domain is also not visible in the structure, we speculate that the SEP domain mediates the interaction to the flexible part of I3 in the space between PP1 and I3 in the p97 pore, which is in line with extra density in this region seen at lower density threshold.

A possible hold‐and‐extract mechanism hypothesized here may more generally apply to protein complex disassembly including ubiquitin and Ufd1‐Npl4‐mediated disassembly of complexes such as Ku70/80 or the MCM2‐7 helicase (van den Boom *et al*, 2016; Mukherjee & Labib, 2019), which would entail direct binding of components of these complexes to the N‐domain. In contrast, a monomeric ubiquitylated substrate protein would not require immobilization for unfolding, which could explain why a direct interaction between the substrate and the p97 N‐domain has not been observed in previous cryo‐EM analyses (Twomey *et al*, 2019). The p97 N‐domains can undergo up and down movements relative to the AAA barrel, driven by the ATP cycle in the D1 domain (Tang *et al*, 2010; Schuetz & Kay, 2016). In our structure, all N‐domains are in the up position which brings the substrate complex close to the central p97 pore. Given that the pulling forces generated by substrate threading in the channel are believed to extract I3 from PP1 and that the SDS22‐PP1 complex is tightly anchored on the N‐domain, additional movement of the N‐domain could contribute to the forces needed to dissociate a protein from its partner.

## Materials and Methods

### Sample purification

p97 and the complex of SDS22, PP1γ, and His‐tagged I3 (SPI complex) were generated in insect cells as previously described (Weith *et al*, 2018). In brief, Sf9 cells were transfected with a pFL vector encoding His‐tagged p97, or His‐tagged I3, or both untagged SDS22 and PP1γ using the dual expression cassette. After virus amplification in Sf9 cells, final protein expression was performed in High Five cells. For the expression of the SPI complex, High Five cells were transduced with both viruses (SDS22 + PP1 and His‐I3).

The SPI complex and the mutant version were purified from cleared High Five cell lysates using a HisTrap FF column (Cytiva) in p97 buffer (50 mM HEPES pH 8.0, 150 mM KCl, 5 mM MgCl_2_, 5% glycerol) with 25 mM imidazole. Protein was eluted directly onto a HiTrap Q HP anion exchange column (Cytiva) with p97 buffer supplemented with 300 mM imidazole. SPI was eluted from the Q HP column using p97 buffer with 1 M KCl and further purified by gel filtration on a Superdex 200 16/600 column (Cytiva) in p97 buffer with 1 mM DTT.

His‐p97 was purified identically to the SPI complex via NiNTA affinity chromatography and anion exchange chromatography. However, the protein was eluted from the Q HP column using a gradient (buffer A: p97 buffer; buffer B: p97 buffer +850 mM KCl), before the buffer was exchanged by repeated concentration and dilution in p97 buffer with 1 mM DTT.

Human p37 was generated from GST‐p37 in *Escherichia coli* BL21 (DE3) as previously described (Weith *et al*, 2018). In brief, cleared bacterial lysate was loaded onto a GSTrap column (Cytiva) using p97 buffer, incubated with PreScission protease for GST‐tag removal, and further purified by gel filtration on a Superdex 200 16/600 column in p97 buffer with 1 mM DTT. Proteins were snap‐frozen in liquid nitrogen and stored at −80°C.

For analysis of sample complex formation, equimolar concentrations of p97, p37, and SPI were separated by size exclusion chromatography on a Superose 6 10/300 increase column in p97 buffer.

For generation of p97‐Q50BpA, an amber stop codon was introduced via QuikChange at position Q50 of p97‐His. Proteins were expressed in bacteria harboring the pEVOL‐pBpF plasmid (Addgene plasmid #31190) as described previously (Weith *et al*, 2018) and purified as described above for wild‐type p97. For cross‐linking, wild‐type p97 or p97‐Q50BpA (1 μM) was mixed with p37 (1 μM) and SPI complex (1 μM) in p97 buffer supplemented with 1 mM DTT and 2 mM ATP. The photoreactive crosslinker was activated by UV irradiation (CL‐1000 crosslinker, 365 nm; Analytik Jena) after spotting the samples spotted on parafilm. Samples were analyzed by western blot with indicated antibodies (Sds22 antibody E‐20, Santa Cruz Biotechnologies; PP1γ antibody C‐19, Santa Cruz Biotechnology; rabbit polyclonal anti‐Inhibitor‐3 (Kracht *et al*, 2020) p97 antibody sc‐57492, Santa Cruz; GST antibody G1160, Sigma) or further processed for mass spectrometry analysis as previously described (Kracht *et al*, 2020).

### GST‐pulldown assays

Binding assays were carried out as described in Weith *et al* (2018). Mutations in p97 were introduced by QuikChange mutagenesis. His‐tagged p97 and GST‐p37 were generated in bacteria. SPI was made in insect cells as described above.

### Cryo‐EM sample preparation

The complexes were reconstituted from frozen aliquots, thawed at room temperature. p97 (5 μM), p37 (4 μM), and SPI (1.5 μM) were mixed in buffer containing 50 mM HEPES pH 7.5, 150 mM KCl, 5 mM MgCl_2_, 2% glycerol, 1 mM DTT, 0.0005% NP40, and 2 mM ADP‐BeF_x_. ADP‐BeF_x_ was prepared freshly from frozen stocks of ADP (0.1 M), BeSO_4_ (1 M), KF (1 M) and MgCl_2_ (1 M) mixed sequentially in that order in relative volumes 1:1:8:1. After mixing, the sample was incubated at room temperature for 10 min and diluted 3:1 in buffer before plunge freezing in liquid ethane using a Vitrobot Mk IV (Thermo Fisher Scientific). 4 μl of sample was loaded on freshly glow discharged 1.2–1.3300 mesh C‐flat™ grids without further support (Electron Microscopy Sciences). The Vitrobot chamber was set to 4°C and 95% humidity, and grids were prepared with a range of blotting time and force, from which the best grids were selected after manual screening.

### EM data collection

Micrograph collection was done at Birkbeck College London using a Titan Krios G3i electron microscope (FEI/Thermo Fisher) operated at 300 kV equipped with a post‐GIF K3 detector and an energy slit 20 eV wide. The images were taken in super‐resolution mode at a nominal magnification of 81,000 with a pixel size of  1.067 Å, dose rate of 15.94 e−/Å^2^/s over 3 s of exposure and a defocus range from −3.3 to −1.8. 6,000 movies were collected using these settings. Preliminary data for this project were obtained in the electron Bio‐Imaging Center (eBIC) at Diamond Light Source using a Titan Krios G3i electron microscope (FEI/Thermo Fisher) operated at 300 kV equipped with a post‐GIF K3 detector. These data were recorded with a 0.845 Å pixel size and 18.5 e−/Å^2^/s over 1.8 s.

### Image processing

For processing, data were resampled to 1.1 Å/pixel. Initial image processing was done in Relion (Scheres, 2012). Movies were motion corrected using Motioncor2 (Zheng *et al*, 2017), and CTF was corrected using Gctf (Zhang, 2016). After extraction and preliminary visual evaluation, 6 million particles were exported to Cryosparc 2 (Punjani *et al*, 2017) for 2D classification. After two rounds of classification, 1.6 million particles were selected for ab initio reconstruction and nonuniform refinement. The particles and resulting map were exported into Relion for further 3D classification and refinement, yielding a subset of 850,000 particles showing signs of substrate occupancy which were selected for further processing. This subset was subjected to 2 cycles of nonuniform refinement and heterogeneous refinement in Cryosparc, resulting in a final selection of 366,000 particles that showed clear densities for the cofactors and substrates. This subset was further processed with Relion for extensive classification. Different masks corresponding to the different possible substrate binding zones were used to obtain subsets showing predominant densities in each of those zones, allowing the study of different populations of SPI‐bound p97. These masks were created using Relion from densities manually extracted from the maps with UCSF Chimera (Pettersen *et al*, 2004). Final maps were refined in Relion following the gold‐standard procedures and presented unmasked and without further postprocessing, aside from the structure focused on p97 alone.

### Model building

The human homology model was created with Swissmodel (Waterhouse *et al*, 2018) based on the structure by (Cooney *et al*, 2019) and fitted in the cryo‐EM map using flexEM (CCPEM; Topf *et al*, 2008). To ensure the best fit, the model was refined against the map in real space first using COOT (Emsley *et al*, 2010), and then PHENIX v1.19 with secondary structure restraints. The model geometry was regularized using the *phenix.geometry_minimization* tool (Afonine *et al*, 2018). Model quality was assessed using TEMPy SMOCf (Farabella *et al*, 2015; Joseph *et al*, 2016) and MolProbity scores (Chen *et al*, 2010; Williams *et al*, 2018). Model‐to‐map FSC was estimated using *phenix.validation_cryo‐EM* tool (Liebschner *et al*, 2019; Appendix Fig S3i). SPI subunit models were based on the structure by (Cooney *et al*, 2019). Figures were made using UCSF Chimera and ChimeraX (Pettersen *et al*, 2021).

### Statistical analysis

No statistical analysis was performed in this study. Western blot experiments were repeated at least three times with similar results, and representative experiments are shown. No blinding was performed.

## Author contributions

**Hemmo Meyer:** Conceptualization; resources; funding acquisition; writing – original draft; project administration; writing – review and editing. **Helen R Saibil:** Conceptualization; resources; data curation; funding acquisition; writing – original draft; project administration; writing – review and editing. **Johannes van den Boom:** Formal analysis; investigation; methodology. **Guendalina Marini:** Data curation; formal analysis.

## Disclosure and competing interests statement

Helen Saibil is a member of the Advisory Editorial Board of *The EMBO Journal*. This has no bearing on the editorial consideration of this article for publication. The authors declare that they have no other competing interests.

## Supporting information



Appendix

Expanded View Figures PDF

Movie EV1

PDF+

## Data Availability

Atomic models have been deposited in the Protein Data Bank (PDB), and cryo‐EM maps have been deposited in the Electron Microscopy Data Bank (EMDB) under the following accession numbers. p97 hexamer: EMD‐15774 (http://www.ebi.ac.uk/pdbe/entry/EMD‐15774); p97 with SPI on subunit B: EMD‐15861 (http://www.ebi.ac.uk/pdbe/entry/EMD‐15861) and 8B5R (https://www.ebi.ac.uk/pdbe/entry/pdb/8b5r); p97 with SPI on subunit C: EMDB EMD‐15847 (http://www.ebi.ac.uk/pdbe/entry/EMD‐15847); p97 with SPI on subunit D: EMD‐15778 (http://www.ebi.ac.uk/pdbe/entry/EMD‐15778); p97 with SPI on subunit E: EMD‐15846 (http://www.ebi.ac.uk/pdbe/entry/EMD‐15846). The mass spectrometry proteomics data have been deposited to the ProteomeXchange Consortium via the PRIDE partner repository with the dataset identifier PXD039939 (http://proteomecentral.proteomexchange.org/cgi/GetDataset?ID=PXD039939).
