# Supplementary material for: Structural basis of ubiquitin‐independent PP1 complex disassembly by p97
Source: EMBO J. 2023 Jun 2;42(14):e113110. doi: 10.15252/embj.2022113110 (PMC10350818; doi:10.15252/embj.2022113110)
Supplement: Supplementary file 1 — Appendix [file EMBJ-42-e113110-s001.pdf]

Appendix

Structural basis of ubiquitin-independent PP1 complex disassembly by p97

Johannes van den Boom[1], Guendalina Marini[2], Hemmo Meyer[1,*], Helen Saibil[3,*]

[1]Molecular Biology I, Center of Medical Biotechnology, Faculty of Biology, University of Duisburg-Essen, Germany
[2]Centre for Structural Systems Biology, Leibniz-Institute of Virology and University Medical Center Hamburg-Eppendorf (UKE), Hamburg, Germany
[3]Biological Sciences, Institute of Structural and Molecular Biology, Birkbeck University of London, UK

*correspondence:
h.saibil@bbk.ac.uk
hemmo.meyer@uni-due.de

Table of contents

Appendix Figures 3

Appendix Fig. S1 3

Appendix Fig. S2 4

Appendix Figures

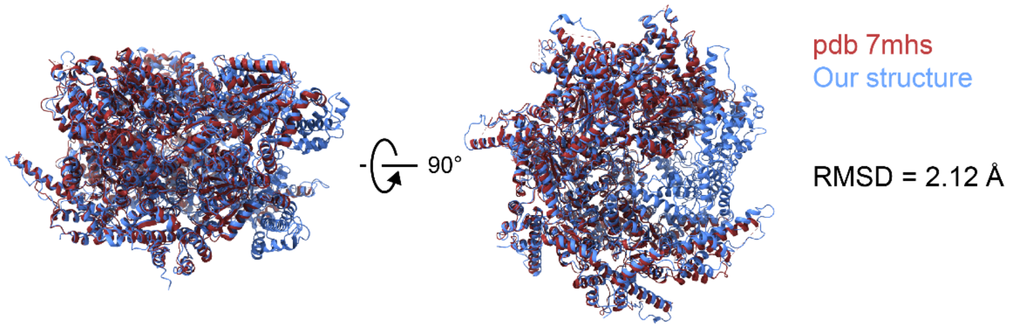

Appendix Figure S1.

Overlay of the aligned atomic models of p97 from our structure and the previously published model (pdb 7mhs; Xu et al. 2022). The two models show high similarity (RMSD = 2.12 Å). N-domains were removed, as coordinates for N-domains and F subunit of the previous structure were not deposited.

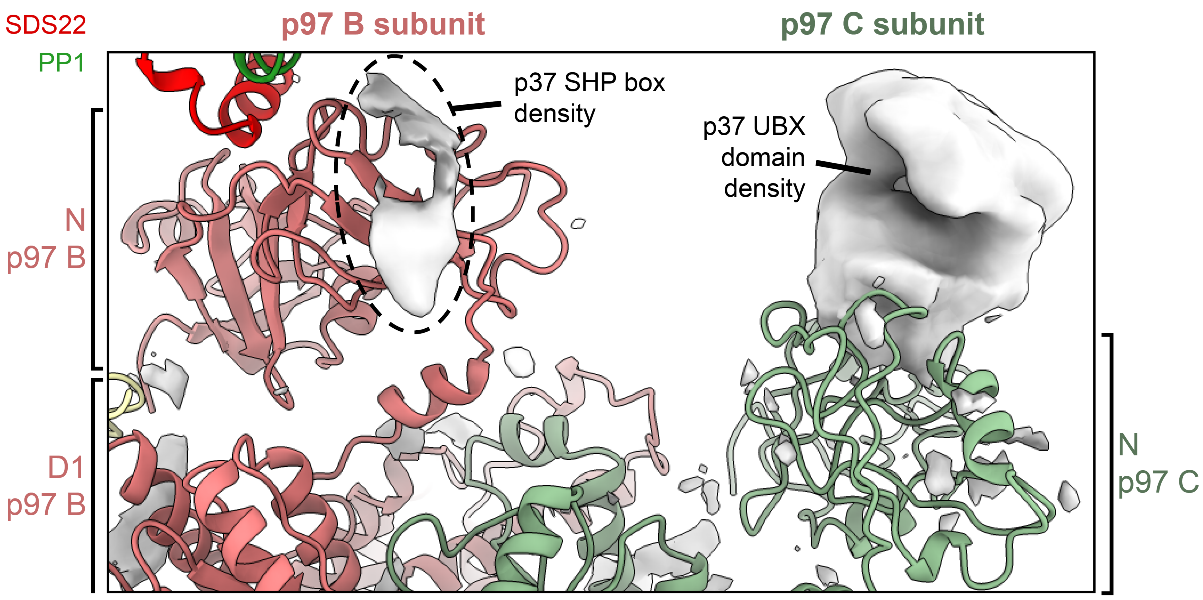

Appendix Figure S2.

Density of p97 and SPI was subtracted from the map to show the additional density (gray) on p97 N-domains of subunits B (left) and C (right) corresponding to the p37 adapter. The additional density on the B subunits corresponds to the SHP box of p37, while the additional density on the C subunit corresponds to the UBX domain of p37. Density threshold of the map is 0.0024.
