# Supplementary material for: Structural basis of ubiquitin‐independent PP1 complex disassembly by p97
Source: EMBO J. 2023 Jun 2;42(14):e113110. doi: 10.15252/embj.2022113110 (PMC10350818; doi:10.15252/embj.2022113110)
Supplement: Supplementary file 2 — Expanded View Figures PDF [file EMBJ-42-e113110-s003.pdf]

Expanded View Figures

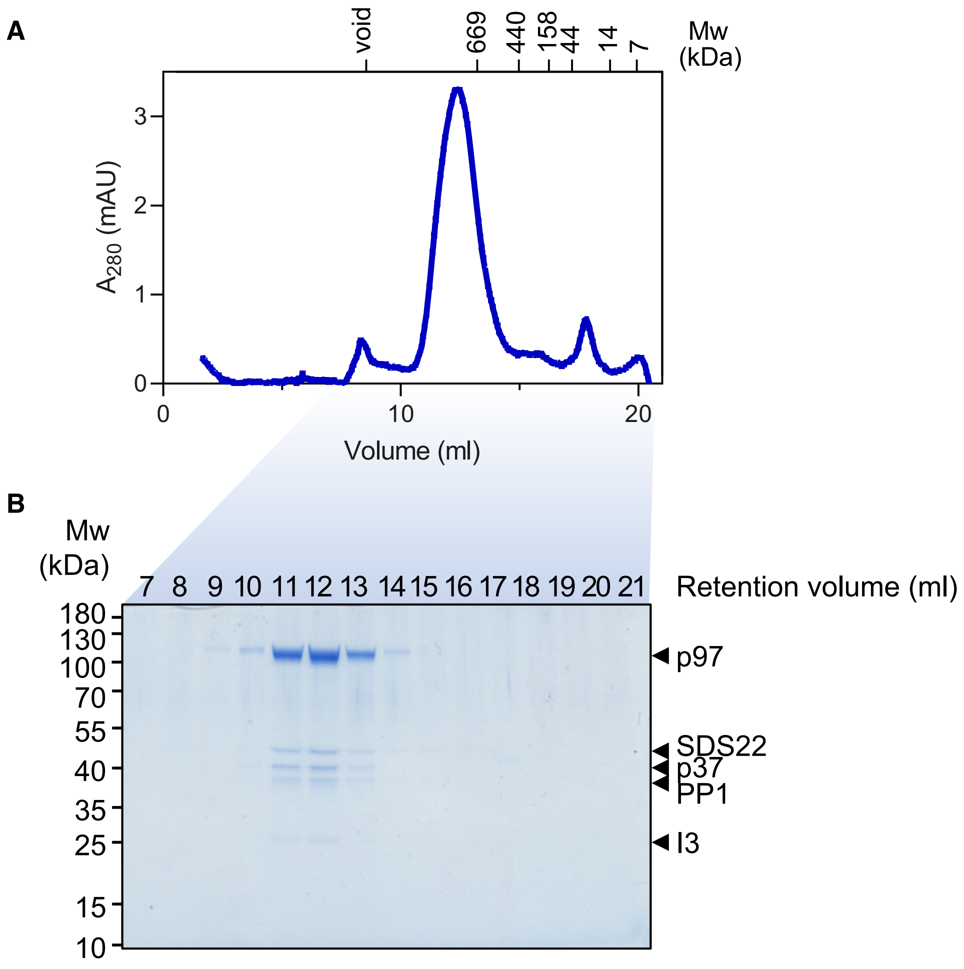

Figure EV1. Purification of the p97-p37-SDS22-PP1-I3 complex.

A Size exclusion chromatogram of equimolar concentrations of p97-p37-SDS22-PP1-I3 complex on a Superose 6 10/300 increase column. Molecular weights of reference standard are indicated.

B Coomassie gel of indicated size exclusion chromatography fractions from (a). Individual components of the complex are labeled.

Figure EV2. Cryo EM data and map validation for p97.

A–C Example raw images from the particle dataset. Scale bar, 500 Å.

D Example 2D class averages, showing some different particle orientations, with some weak substrate densities adjacent to the stronger p97 features. The box size is 352 × 352 Å.

E Gold-standard Fourier shell correlation (GSFSC) calculated during refinement without and with mask. The resolutions were determined at FSC = 0.143 (dotted line).

F Plot of particle orientations.

G Local resolution was calculated from the half-map and colored according to the scale on the side. p97 subunits A–F labeled. For this map 866,937 particles were used.

▶

© 2023 The Authors

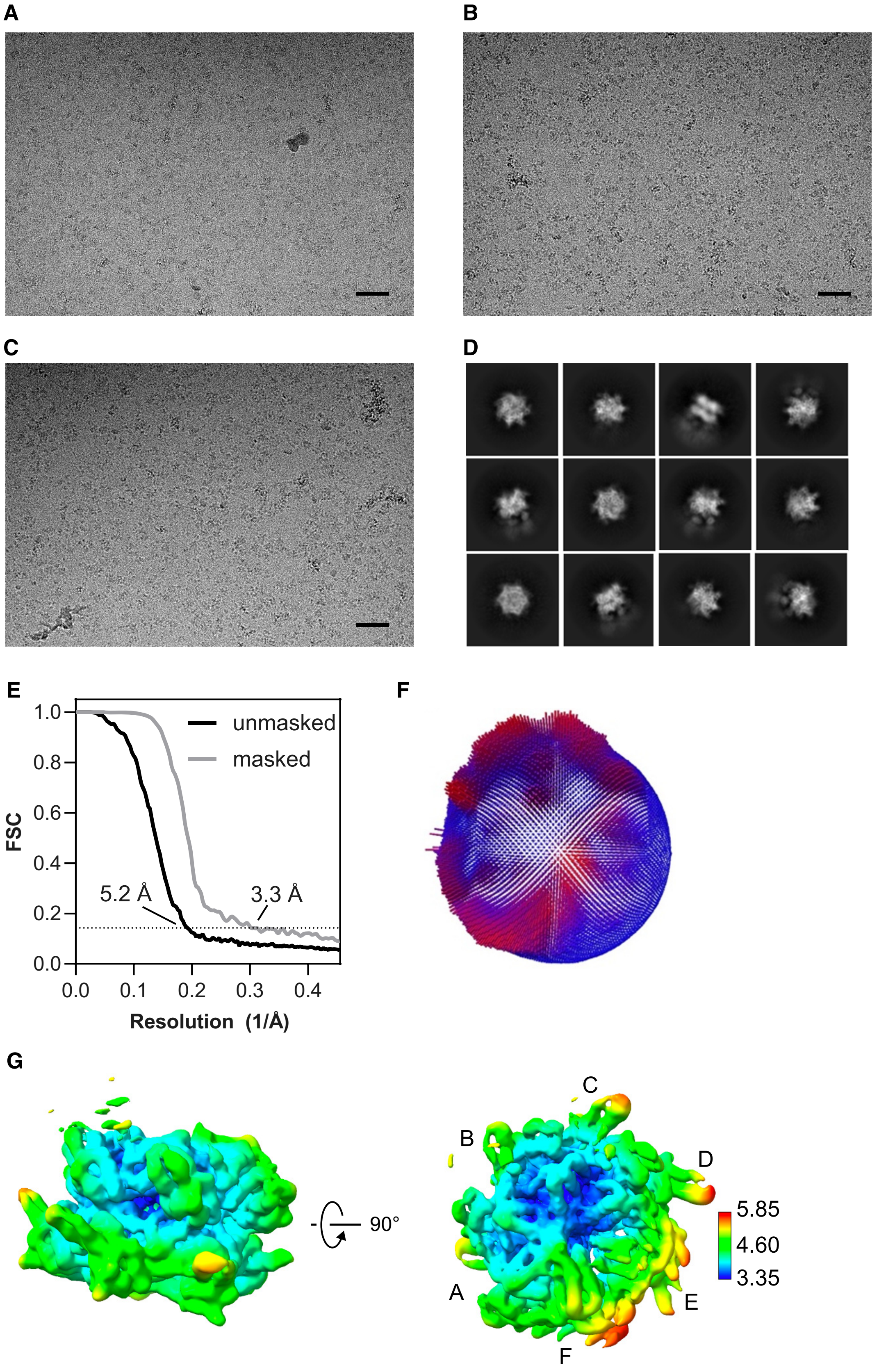

Figure EV2.

 © 2023 The Authors

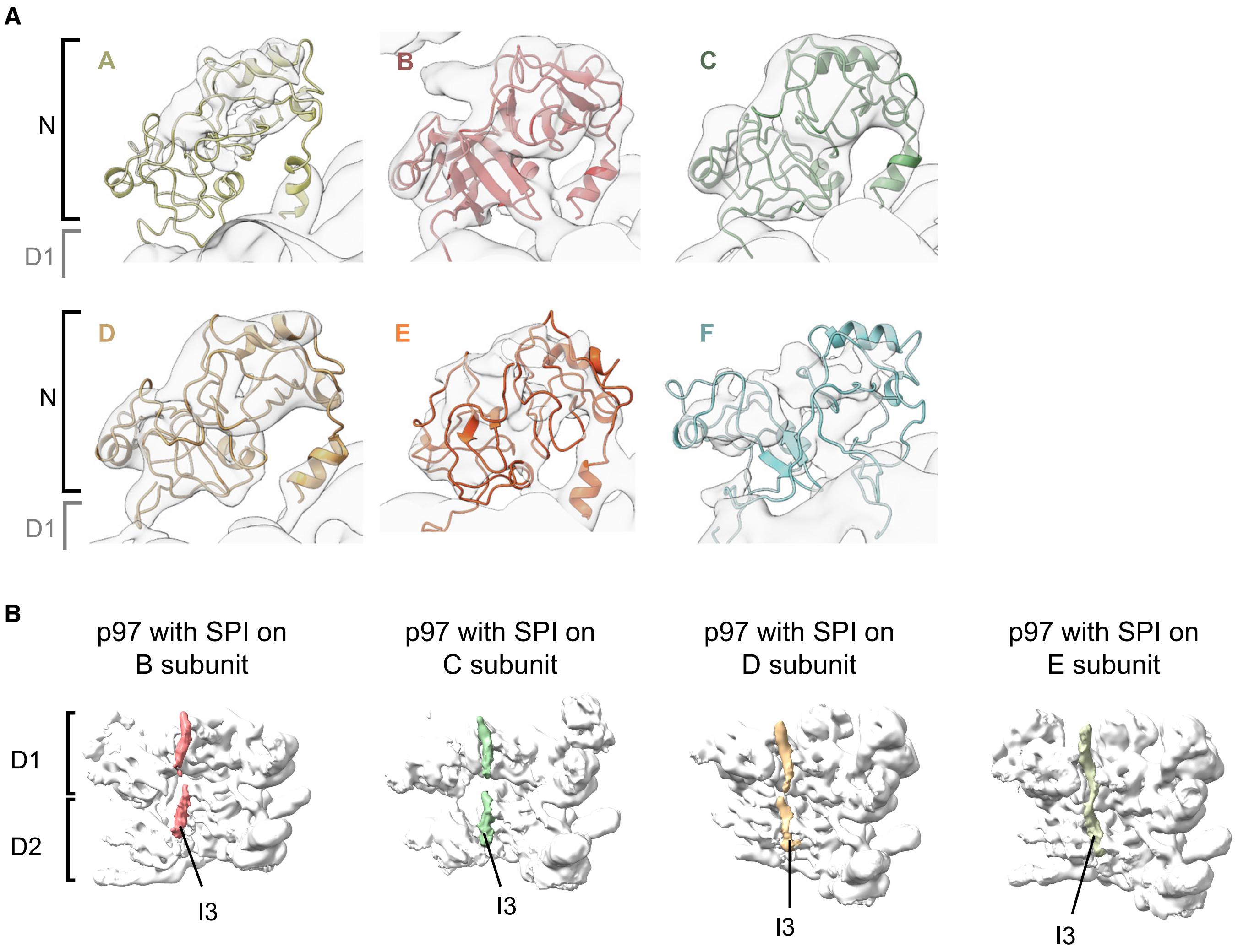

Figure EV3. p97 N-domains and density of threading substrate polypeptide.

A Zoomed-in view of p97 N-terminal domains seen on the map with SPI bound to subunit B with the atomic models docked in. All six N-domains of the p97 hexamer are clearly defined by the density to be in the "up" conformation.

B Views of the I3 substrate in the p97 pore of the maps with SPI bound on p97 subunits B, C, D, and E as indicated. Three protomers in the front of the p97 hexamer were removed for clarity.

Figure EV4. Validation information for p97 complex maps and model fitting.

A Local resolution of the p97 map with SPI bound on subunit B colored according to the scale below. The resolution scale mainly serves to show the relative resolutions of different parts of the structure. The absolute values for the mobile N and substrate domains appear too optimistic.

B–E Gold-standard Fourier shell correlation (FSC) plot for the p97 map with SPI bound on the B subunit (B), C subunit (C), D subunit (D), and E subunit (E). Resolutions were determined at FSC = 0.143 (dotted line).

F Particle orientation distribution and particle numbers indicated below for the p97 map with SPI bound on the B, C, D, or E subunit.

G SMOCf score plots are shown per residue for every subunit. Dashed lines indicate average values.

H Plot of the map-to-model FSC with and without mask showing similar FSC curves and resolutions. Resolutions were determined at FSC = 0.143 and 0.5 (dashed lines), as indicated.

I Summary of the average CC and SMOCf scores for each subunit. All FSCs were calculated without masking.

© 2023 The Authors

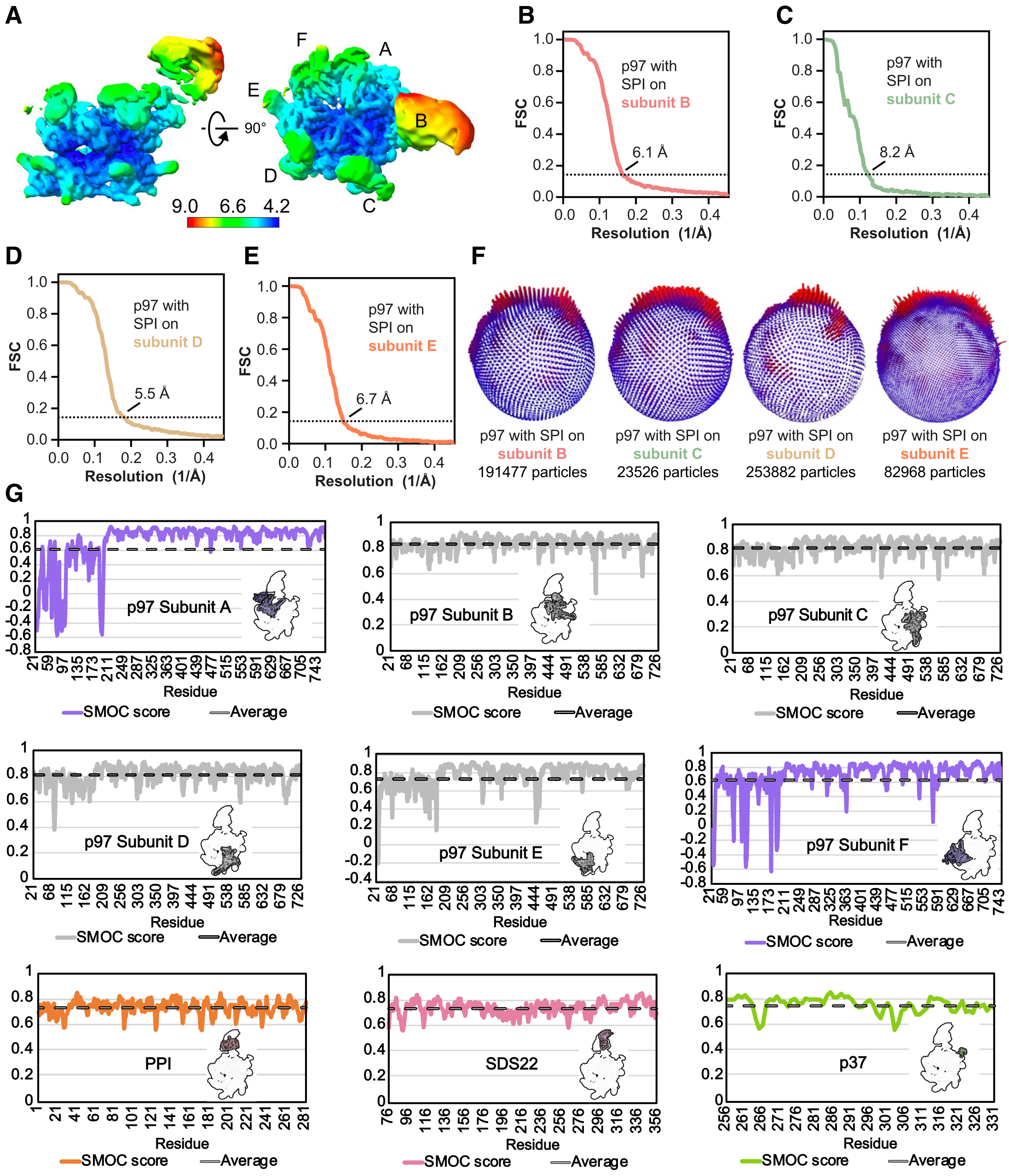

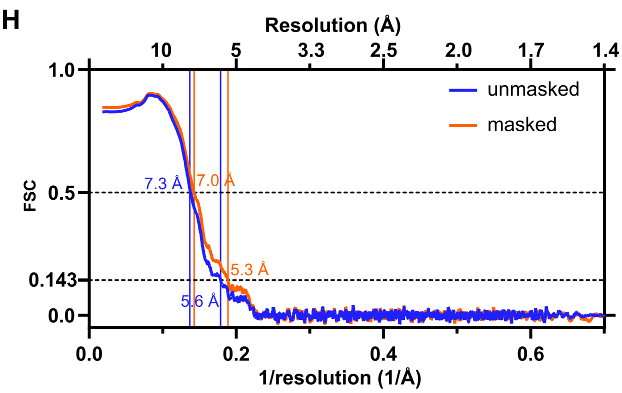

I

Subunit	Avg CC score	Avg SMOCf score
p97 A	0.82	0.63
p97 B	0.84	0.84
p97 C	0.84	0.82
p97 D	0.83	0.81
p97 E	0.80	0.73
p97 F	0.77	0.64
PPI	0.78	0.74
SDS22	0.79	0.74
p37	0.80	0.75

Figure EV4.

 © 2023 The Authors

Figure EV5. Biochemical analysis of the p97-SDS22 interface.

A p97-Q50BpA cross-link sample from Fig 4B was analyzed by mass spectrometry. One peptide with an FDR <5% was detected. The BpA at amino acid position 50 (× in the sequence) in p97 is cross-linked to a peptide of SDS22 covering residues 336–341. Identified y ions are indicated (┌).

B Western blot analysis of cross-links between p97 with BpA crosslinker at residue Q50 and SDS22 in the presence of different nucleotides (2 mM) as indicated. p97-Q50BpA was incubated with p37, SPI and different nucleotides before cross-linking by UV irradiation or mock treatment as indicated. p97-SDS22 cross-link, p97 interprotomer cross-links (p97 multimers), as well as non-cross-linked p97 and SDS22 are labeled. γS denotes ATPγS.

C Views of the interaction site between SDS22 and the p97 N-domain groove. Mutated residues in p97 chosen for proximity to the SDS22-p97 contact site are labeled. Both G54 and Y143 are at 5.1 Å distance (Cα-Cα distance) from the SDS22 helix.

D GST-pulldown binding assays using purified GST or GST-p37, SPI, and p97 variants with the chosen mutations as indicated were carried out in the presence of ATP. Western blot with indicated antibodies. Note that the mutations largely reduce SPI but not p37 binding.

E Views of the interaction site between SDS22 and the p97 N-domain groove. Mutated residues in SDS22 chosen for proximity to the SDS22-p97 contact site are labeled.

F GST-pulldown binding assays using purified GST or GST-p37, without or with p97 and with SPI or SPI variant with the chosen mutations (R341A + K342A, labeled 2A) as indicated were carried out in the presence of ATP. Western blot with indicated antibodies. Note that the mutations abolish SPI binding to p97-p37.

© 2023 The Authors

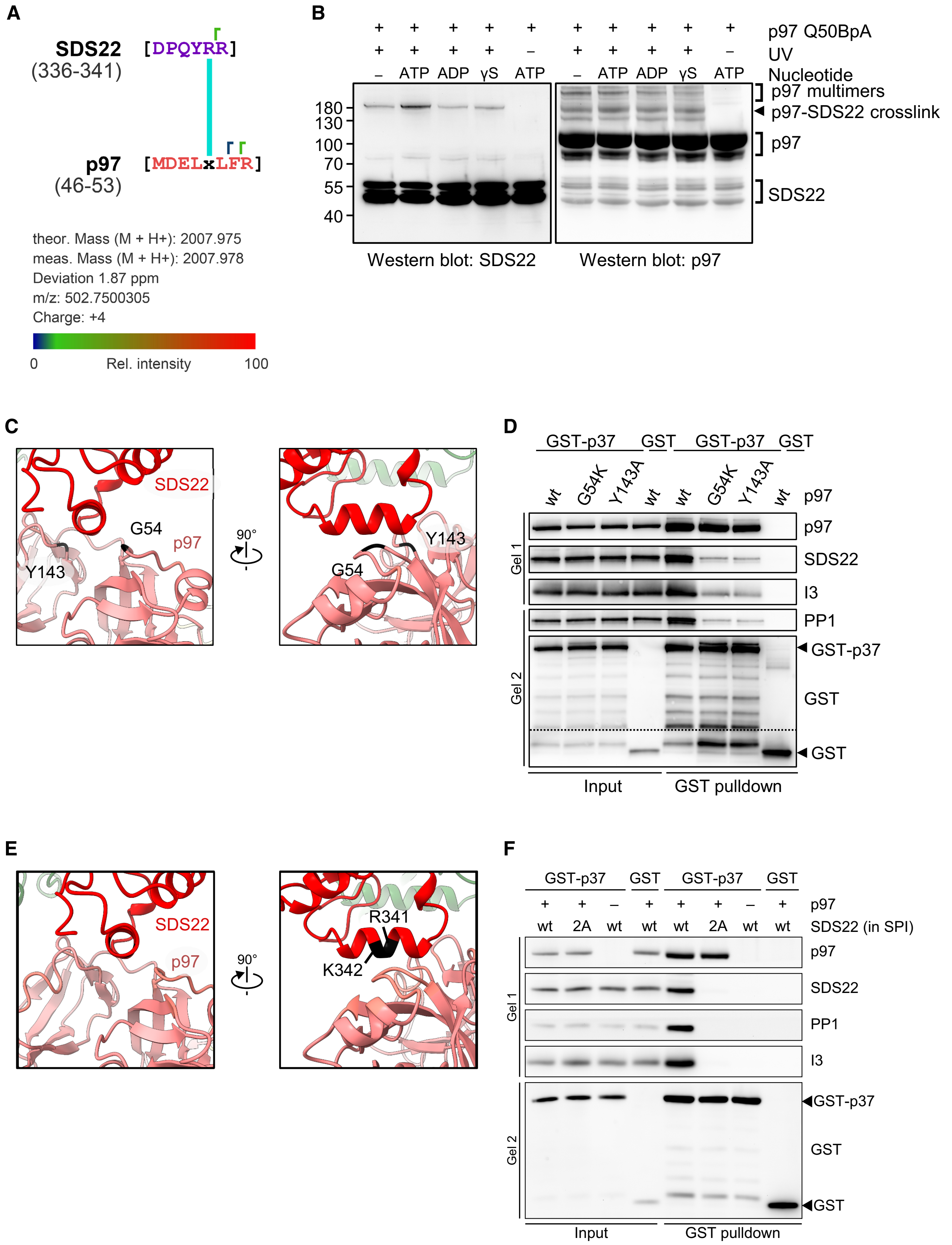

Figure EV5.

© 2023 The Authors
