# Supplementary material for: Structural basis of ubiquitin‐independent PP1 complex disassembly by p97
Source: EMBO J. 2023 Jun 2;42(14):e113110. doi: 10.15252/embj.2022113110 (PMC10350818; doi:10.15252/embj.2022113110)
Supplement: Supplementary file 3 — Movie EV1 [file EMBJ-42-e113110-s004.zip › Movie EV1 Legend.docx]

**Movie EV1.**

Model of the p97-p37-SPI complex. Views of the complex with SPI bound on the B subunit of p97. Proteins are coloured as in Figure 6. The movie was generated using ChimeraX 1.3 and Adobe Premiere Pro 2022.
