# Supplementary material for: Structural basis of ubiquitin‐independent PP1 complex disassembly by p97
Source: EMBO J. 2023 Jun 2;42(14):e113110. doi: 10.15252/embj.2022113110 (PMC10350818; doi:10.15252/embj.2022113110)
Supplement: Supplementary file 5 [file EMBJ-42-e113110-s005.pdf]

THE EMBO JOURNAL

Structural basis of ubiquitin-independent PP1 complex disassembly by p97

Hemmo Meyer, Helen Saibil, Johannes van den Boom, and Guendalina Marini
DOI: 10.15252/embj.2022113110

Corresponding author(s): Hemmo Meyer (Hemmo.Meyer@uni-due.de) , Helen Saibil (h.saibil@bbk.ac.uk)

Review Timeline:

Submission Date:	21st Nov 22
Editorial Decision:	6th Jan 23
Revision Received:	16th Feb 23
Editorial Decision:	19th Apr 23
Revision Received:	12th May 23
Accepted:	22nd May 23

Editor: Hartmut Vodermaier

Transaction Report:

(Note: With the exception of the correction of typographical or spelling errors that could be a source of ambiguity, letters and reports are not edited. Depending on transfer agreements, referee reports obtained elsewhere may or may not be included in this compilation. Referee reports are anonymous unless the Referee chooses to sign their reports.)

Prof. Hemmo Meyer
Universitat Duisburg-Essen
Faculty of Biology
Institute of Molecular Biology 1
Campus Essen
Essen 45117
Germany

6th Jan 2023

Re: EMBOJ-2022-113110
Structural basis of a hold-and-extract mechanism underlying ubiquitin-independent PP1 complex disassembly by p97

Dear Hemmo and Helen,

Thank you again for submitting your structural study on PP1 complex disassembly by p97 to The EMBO Journal, and apologies for the delay in getting back to you with reviewer comments due to the end-of-the-year holidays. Your study has now been seen by three expert referees, whose reports are copied below. As you will see, while they all acknowledge the importance of this work as well as potentially interesting findings, they raise a number of overlapping concerns that would need to be satisfactorily addressed prior to publication. In particular, the referees point out that not all conclusions are at present strongly justified by the structural data, and that more of the supportive biochemical and structural data should be deposited and/or provided to the referees for better assessment.

Should you be able to adequately respond to the various issues noted in all three reports, I would be happy to consider a revised version further for EMBO Journal publication. Since it is our policy to allow only a single round of major revision, I would however encourage you to contact me with a tentative response letter and revision plan outlining how you might address each point already during the early stage of the revision, so that we could further discuss the essential revisions and how they might be achieved. I should add that we could also offer extension of the default three-months revision period if needed, with our 'scooping protection' (meaning that competing work appearing elsewhere in the meantime will not affect our considerations of your study) remaining of course valid also throughout this extension.

Detailed information on preparing, formatting and uploading a revised manuscript can be found below and in our Guide to Authors. Thank you again for the opportunity to consider this work for The EMBO Journal, and I look forward to hearing back from you in due time.

With kind regards,

Hartmut

Hartmut Vodermaier, PhD
Senior Editor, The EMBO Journal
h.vodermaier@embojournal.org

*** PLEASE NOTE: All revised manuscript are subject to initial checks for completeness and adherence to our formatting guidelines. Revisions may be returned to the authors and delayed in their editorial re-evaluation if they fail to comply to the following requirements (see also our Guide to Authors for further information):

1) Every manuscript requires a Data Availability section (even if only stating that no deposited datasets are included). Primary datasets or computer code produced in the current study have to be deposited in appropriate public repositories prior to resubmission, and reviewer access details provided in case that public access is not yet allowed. Further information: embopress.org/page/journal/14602075/authorguide#dataavailability

2) Each figure legend must specify
- size of the scale bars that are mandatory for all micrograph panels
- the statistical test used to generate error bars and P-values
- the type error bars (e.g., S.E.M., S.D.)
- the number (n) and nature (biological or technical replicate) of independent experiments underlying each data point
- Figures may not include error bars for experiments with n<3; scatter plots showing individual data points should be used instead.

3) Revised manuscript text (including main tables, and figure legends for main and EV figures) has to be submitted as editable text file (e.g., .docx format). We encourage highlighting of changes (e.g., via text color) for the referees' reference.

4) Each main and each Expanded View (EV) figure should be uploaded as individual production-quality files (preferably in .eps, .tif, .jpg formats). For suggestions on figure preparation/layout, please refer to our Figure Preparation Guidelines: http://bit.ly/EMBOPressFigurePreparationGuideline

5) Point-by-point response letters should include the original referee comments in full together with your detailed responses to them (and to specific editor requests if applicable), and also be uploaded as editable (e.g., .docx) text files.

6) Please complete our Author Checklist, and make sure that information entered into the checklist is also reflected in the manuscript; the checklist will be available to readers as part of the Review Process File. A download link is found at the top of our Guide to Authors: embopress.org/page/journal/14602075/authorguide

7) All authors listed as (co-)corresponding need to deposit, in their respective author profiles in our submission system, a unique ORCiD identifier linked to their name. Please see our Guide to Authors for detailed instructions.

8) Please note that supplementary information at EMBO Press has been superseded by the 'Expanded View' for inclusion of additional figures, tables, movies or datasets; with up to five EV Figures being typeset and directly accessible in the HTML version of the article. For details and guidance, please refer to: embopress.org/page/journal/14602075/authorguide#expandedview

9) Digital image enhancement is acceptable practice, as long as it accurately represents the original data and conforms to community standards. If a figure has been subjected to significant electronic manipulation, this must be clearly noted in the figure legend and/or the 'Materials and Methods' section. The editors reserve the right to request original versions of figures and the original images that were used to assemble the figure. Finally, we generally encourage uploading of numerical as well as gel/blot image source data; for details see: embopress.org/page/journal/14602075/authorguide#sourcedata

At EMBO Press, we ask authors to provide source data for the main manuscript figures. Our source data coordinator will contact you to discuss which figure panels we would need source data for and will also provide you with helpful tips on how to upload and organize the files.

Further information is available in our Guide For Authors: https://www.embopress.org/page/journal/14602075/authorguide

In the interest of ensuring the conceptual advance provided by the work, we recommend submitting a revision within 3 months (6th Apr 2023). Please discuss the revision progress ahead of this time with the editor if you require more time to complete the revisions. Use the link below to submit your revision:

Link Not Available

Referee #1:

In this manuscript, van den Boom et al. present the first cryo-EM structures of the AAA+ ATPase p97 bound to the p37 adapter and the SPI complex, consisting of the protein phosphatase 1 (PP1), its binding partner SDS22, and the PP1 inhibitor I3. A major new finding lies in the identification of a previously unknown direct interaction between PP1-bound SDS22 and p97's N-domain, where a short C-terminal helix of SDS22 docks into the groove between the Nn and Nc subdomains of p97 to position SPI for I3 extraction. The authors also observed extra density in the central channel of the ADP-BeFx-bound p97 that likely represents the N-terminus of I3 engaged by the translocation machinery, with the ATPase ring forming a spiral arrangement that is consistent with previously solved structures of substrate-engaged AAA+ motors. Furthermore, the p37 adapter was founds to interact only through its SHP box with the SPI-bound p97 N-domain, while its UBX domain contacts a neighboring N-domain. The presented structures thus provide important new insight not only into the mechanisms that underlie the targeting of SPI to p97 for I3 stripping and PP1 activation, but also into general principles of ubiquitin-independent substrate processing mediated by UBX-domain containing adapters.
The newly discovered SPI interactions with p97's N-domain are well supported by the docked crystal structure of the SDS22-PP1 complex and are further validated through convincing mutational studies. Similarly, thanks to a previously solved crystal structure of the complex between p37's SHP-box motif and p97's N-domain it was possible to identify a potential SHP-box interaction with the SPI-bound N-domain, despite the rather low resolution of the reconstructions in these areas.

Other aspects of this manuscript, however, are less solid and more handwavy, especially regarding PP1/I3/p37 interactions. The manuscript will therefore benefit from revisions in the form of further data analyses, rewriting of particular sections, and some additional experiments, as outlined in detail below.

Main points:
1) The limited resolution represents a significant challenge in reliably assigning extra densities observed for p37/SPI-bound p97, and the authors seem to overestimate the resolution of their reconstructions. Contrary to the claims in Extended Data Figs. 1c and 3a, the resolution of SDS22, the p97 N-domains, and p37's UBX domain is not in the 7-9 Angstrom range, as that would allow a clear detection of secondary structures. Instead, it seems to lie in the 15 - 20 Angstrom range, which only reveals overall envelopes.
Higher flexibility and potentially alternative conformations appear to limit the resolution in the more peripheral areas of the complex. Have the authors considered re-extracting particles with a focused refinement around the N-terminal domains in order to identify different conformational states with higher resolution?
Without the existing crystal structure of the SHP-box bound to the p97 N-domain, the extra density shown as a small white blob in Fig. 3c would not be sufficient for an assignment to p37. The authors should specify how this extra density was identified, e.g. by creating a difference map using other N-domains in the p97 hexamer.

2) Interpreting the unassigned densities 1 and 2 in Fig. 4 is even more challenging. It is certainly conceivable that density 1 near the PP1 active site represents a C-terminal part of I3, but the assignment for density 2 is much less convincing. The authors propose that this extra density 2 originates from I3's RVXF motif, ignoring that it is observed several Angstroms away from the apparently empty RVXF-binding site on PP1. In the discussion, it is stated that "we cannot exclude that the disordered N-terminus of SDS22 also contributes to this density", which highlights the need for either further data analyses and classification to reach a higher resolution or the generation of mutants, for instance a N-terminal truncation of SDS22.
In addition, Fig. 2a shows a large, blobby density on SPI bound to the C subunit of p97, which is acknowledged by the authors but not further explained.

3) The authors propose a hold-and-extract mechanism for the release of I3 from SDS22-PP1 that relies on the assumption that the observed architecture and SPI's interaction with p97 are maintained during active pulling on I3. However, there is no experimental evidence supporting this model, as the reconstructions represent a true initiation complex, in which SPI and p37 interact with hydrolysis-inactive p97 in a state prior to ATPase-driven force application. Whether SDS22-PP1 is held in the same or similar N-domain-bound position during I3 extraction depends on how the strengths of I3 interactions with various regions of PP1 compare to the interaction between the short C-terminal helix of SSD2 and the p97 N-domain cleft, for which there are no experimental data. Although it is possible that an extraction complex looks similar to the presented structure, it is equally conceivable that mechanical tugging on I3 dislodges SDS22-PP1 from its binding site on the N-domain and pulls it against the p97 pore for I3 extraction. The authors should consider this and qualify their claims about an extraction mechanism, including in the title.
Similarly, it is not quite correct that the structures "show p97 ... in the act of translocating a segment of I3", as stated in the first paragraph of the results. Translocation is ATP-hydrolysis dependent, whereas the presented structures originated from SPI binding to a hydrolysis-dead p97 in complex with ADP-BeFx.

4) Based on previous structures of substrate-bound p97 and Cdc48, the central channel accommodates about 20 residues of a substrate polypeptide. If the far N-terminal region of I3 adopts a hairpin conformation, as proposed by the authors, the number of I3 residues inside the channel may be at least 25. This leaves only 15 residues to span the 60 A distance between the RVXF binding site on PP1 and the entrance to the p97 pore, which would be quite a stretch, especially if the authors suggest additional interactions of this I3 region with p97's SEP domain (schematic in Fig.5). Instead, SEP domain interactions may guide I3 toward and into the p97 pore and occur prior to the channel-engaged state observed in the presented structures. The authors should consider these aspects, distances, and number of available I3 residues in more detail. There is also a discrepancy between Fig. 5, which indicates a distance of 60 A between the pore and extra density 2, and the movie, where this distance is given as 50 A.

5) The authors state in the discussion that the interface of SDS22-PP1 with p97 "is not solely conferred by the adapter" and that "multivalent interactions of adapter, the p97 N-domain and subunits of the SPI complex appear to tightly dock and hold SDS22-PP1 on the p97 N-domain". However, based on the presented structures, the p37 adapter appears to not at all contribute to the interface of SDS22-PP1 with p97 or a tight docking, as there are no p37-SPI interactions observed in these structures. If p37 stabilized SPI, one would expect some density to be resolved. It is possible that the stabilization of the SPI-p97 complex by p37 originates from linking two neighboring N-domains through their interactions with the UBX domain and SHP-box, and it could be helpful to assess whether the two p37-contacted N-domains show any conformational differences compared to the other four N-domains within the hexamer.
Overall, none of the previously proposed interactions between p37 and PP1 or I3 are resolved in these structures, which limits the conclusions that can be drawn about p37's role in SPI processing by p97.

Minor points:
1) SPI is observed bound to various p97 subunits (B, C, D, and E) that adopt different position within the spiral conformation of the hexamer. The authors present this in a circular arrangement in Fig. 2b, where different states are linked by arrows. This does not seem ideal and may be misinterpreted as SPI "rotating" on top of the p97 hexamer. There may be better ways to show that

1) during initiation SPI can bind p97 subunits irrespective of their position in a spiral arrangement and 2) these states may be equivalent to conformations expected during a subsequent hand-over-hand threading of I37 through the pore.

2) The legend for Fig. 4 mentions "side, front, top, and bottom views", but only 3 views are shown.

3) Additional bands detected in the Western blots for SDS22 and p97 in Extended Data Fig. 4b should be explained/labeled. In the legend for Extended Data Fig. 4c, it needs to say "p97-Q50BpA crosslink sample from (b)" not "(d)".

4) The legend for Extended Data Fig. 5 does not specify how individual components in panels b and c were detected (presumably by Western blotting).

Referee #2:

The manuscript by Van den Boom et al deals with the AAA ATPase p97, known to regulate diverse cellular processes. It acts as an unfoldase in the ubiquitin-proteasome system, disentangling multi-protein complexes in a very specific and tightly controlled manner. Moreover, p97 is known to work in conjunction with adaptors required to direct its potentially damaging activity towards certain substrates. The Ufd1-Npl4 adaptor that binds ubiquitin and presents it to the AAA1 ring, is critical to remodel Ub-marked substrates. Aside this well-characterized mechanism, p97 can also recruit substrates in a ubiquitin-independent manner, a mechanism often used to separate multi-protein complexes into smaller units. However, the molecular basis of how single subunits are extracted while preserving the substrate's core is little understood. To address this point, the authors reconstituted the complex between p97, the adaptor p37 and the tri-partite substrate complex SPI (SDS22, PP1, I3), one of the best characterized clients. The authors determined the cryo-EM structure of the p97:SPI complex in presence of the ATP analog ADP-BeFx. Remarkably, p97 directly binds to the substrate, i.e. to the SDS22 subunit, and not as commonly assumed via its p37 adaptor protein. Instead, it is suggested that the adaptor takes part in the disassembly mechanism, assisting in the extraction and unfolding of the I3 subunit from the SPI complex. The cryoEM structure also provides further insight into adaptor binding to p97. It is shown that p37 binds to p97 in an extended conformation, with its two binding motifs spanning two neighbouring N-domains of the AAA hexamer. Together, multivalent interactions between p37, PP1, SDS22 and p97 N-domains result in tight binding of the SDS22 and PP1 subunits, required to extract the I3 module from the SPI core. Biochemical data support the major findings inferred by the structural model, leading the authors to propose a "hold-and-extract" mechanism of how p97 remodels multiprotein complexes. The disassembly mechanism should be broadly relevant for regulating many multiprotein complexes and is thus of great interest. In conclusion, the manuscript offers exciting insight into the mechanism of p97 substrate recognition and processing. It's a very nice study, and I have only a few points that the authors could address, if experimentally feasible:

1) The identified interaction between SDS22 and p97's N-domain relies on a specific interface, characterized by complimentary protein shapes and electrostatic properties. Given this specific SPI-p97 contact, how do the authors translate their findings to other multiprotein complexes? Could the identified receptor site in p97 serve as general substrate binding patch, e.g. via charge-charge interactions? Or do other clients undergo different yet specific contacts with the N-domain? I was also wondering whether the authors tried to model client complexes with the p97 N-domain, using AF2? Having SPI as positive control, such in silico screening may provide a quick estimate of further candidate complexes.
2) The structural analysis of the SPI:97 complex is focused on the state where client is bound to subunit B. It seems to be the subunit where the SPI core is best defined but presenting only one of several states. I have questions to the remaining classes: Can the authors comment on the particle distribution of the separate states, which states were most/least populated in EM recordings? Can the authors comment on the unassigned density, attributed to I3 - e.g. is it seen also in other states, even if at lower resolution? Could these different states present a translocation movie, visualizing a gradual discharge of I3 from the SPI complex and/or changes in the density in the central pore (via A-B-C-D-E-F separated states)? It would be great, if the authors could briefly comment on the mechanistic relevance of the separated structures.
3) The pBpA crosslinking data look extremely nice. I would suggest moving this figure from the SI to the main part.
4) Taken the geometry of p37 binding into account - could the SHP-linker-UBX interactions with adjacent p97 N-domains be validated? E.g. shortening the linker by a few residues and preventing its simultaneous interaction by two N-domains? To better illustrate the coupled binding of p37, I would suggest showing the SHP-linker-UBX densities in one figure panel.
5) The location of I3 was deduced by "two regions of additional density that are not filled by PP1 or SDS22". Could this point be backed up by functional data? XLMS data or further cryoEM analysis of one of the other p97:SPI states would strengthen this argument.

Minor points
(175-190) The authors' make some claims about the positioning of the p37 adaptor, where they conclude p37 is bound in an extended conformation. The reasoning is fitting, but should ideally also comment on the possibility of more than one copy of p37 being bound to p97, rather than both binding sites belonging to the same p37 molecule, and clarify the reasons why they believe it's the same p37 molecule.
(297) Authors claim that the hold-and-extract mechanism is shown in the present study. However, the role of p37 to hold SPI in place, preventing it to get pulled into the unfoldase, was not fully validated by biochemical data. It may be helpful to state that the

„holding" mechanism is rather a hypothesis than a claim. Alternatively, further biochemical evidence is required to discriminate the presenting role of p37 from the alternate hypothesis that the adaptor helps to increase affinity for SPI. In fact, Extended Data Fig. 5 (615-625) suggest a strong effect on the affinity for substrate rather than spurious unfolding due to lack of „holding".
(555-559) I would encourage the authors to submit the raw micrographs and/or the motion-corrected micrographs to an appropriate repository (EMPIAR). It is unclear whether masks and half-maps were included in the EMDB entries as additional maps - if not, I would encourage including them. Moreover, please upload the cross-linking MS data to the PRIDE repository.
(Extended data figure 5, panel b) - the composite image shows Western blots for multiple proteins, some of which have very similar size (SDS22 41 kDa, PP1 37 kDa) and could not have been separated by cutting the membrane in bands of appropriate size. There is no information about whether this data comes from membranes that were stripped and re-treated with a different antibody, or multiple gels were run. Would be reassuring if this information would be included in the methods, or if it was otherwise noted how cross-signalling was avoided.
(Figures) The complementary charges of SDS22 and p97 should be shown, highlighting the two mutated Lys/Arg residues.

Referee #3:

Comments for the manuscript entitled " Structural basis of a hold-and-extract mechanism underlying ubiquitin-independent PP1complex disassembly by p97 by van den Boom et al.

In this manuscript, the authors present a cryo-EM structure of p97 in complex with p37 and PSI formed by incubating relevant components in the presence of ADP-BeFx. The structure shows that PP1 and its partners SDS22 and inhibitor-3 (I3) are loaded tightly onto p97 via a direct contact of SDS22 with the p97 N-domain. The role of p37 adapter is to bridge two adjacent p97 N-domains underneath PSI. The structure also shows that the hexameric p97 is in a helical configuration with all 6 N-domains in the up conformation. The structural data suggested that a stretch of I3 is threaded into the central channel, while other elements of I3 are still attached to PP1. A hold-and-extract mechanism for p97-mediated disassembly is proposed.

The work is novel in providing new structural and functional insights into the function of p97. That said, following issues in the manuscript deserve attention from the authors.

Major issues

(1) There is a lack of details in describing experimental procedures used in this work, especially in sample preparation. At Line 95 (L95) it says that "The resulting p97-p37-PSI complex was purified by size exclusion chromatography and analyzed by cryo-EM." No details provided. This reviewer requests to see the size exclusion profiles and 1 or 2 EM images to be included in the supplementary data. This will help readers to know more about complex elution volume, stability, and how the complex looks on EM grids. A SDS-PAGE of the peak fraction in the SEC profile containing all components would also be very helpful. L129: It is also good to know how many classes requested for 2D or 3D classifications? A figure for 2D class averages is needed to show different orientations. Are there any orientation preference among these different 3D classes? It is also not clear whether the structure presented in Figure 1 was produced before the 3D classification or is one of the 4 classes.

(2) Along the same line as in (1), it would be interesting to know why ADP*BeFx was used in this experiment instead of ATPyS reported in their previous experiments (van den Boom et al., NSMB 2021). How relevant is this change in using ADP•BeFx instead of ATPgS with respect to the reaction cycle of the unfolding process should be discussed.

(3) The paper concludes that all N domains are in the Up conformation without showing experimental data. This reviewer has not seen any p97 structures with good densities for N-domain or in uniform up conformation for wild-type p97. If the authors are so confident about the N-domain conformation, they should show it. Previous works showed the correlation between the conformation of N domain and the cognate D1 nucleotide state. Can the authors make a conclusion on the nucleotide-binding state for each subunit in the D1 and D2 domains? This question becomes important considering the proposed p37 binding between N-domains of neighboring subunits.

(4) It would be interesting to compare current structure with previously published ones. Just by visual inspection, it seems quite different. Can a comparison be made? L105: It was mentioned that subunit F is disordered, bridging subunits A (highest position) and E (lowest position), referencing Fig.1 and Extended Data Fig. 1. In Figure 1, it is not obvious at all that the F subunit is disordered. In the Extended Figure 1, it does not even mark where the subunit F is. Also, in general how does one distinguish between flexibility and lower occupancy?

(5) L133: please verify that there is no detected class (even at low resolution) with SPI bound to subunit A and F. Or it's just extremely low particle count which can be neglected. L138: The sentence "Subunit B shows ...". This reviewer doubts that this statement is correct. First of all, SPI density looks best when bound to subunit B and D, whereas C and E are a bit poor. However, this can be influenced by the number of particles in each class. B 191,477; C 23,526; D 253,882; E 82,968. So the authors should either focus on the number of particle in each class to draw conclusion that SPI binds to subunit B and D most of the time (occupancy) or normalize the particle size and analyze the resolution and density quality. Furthermore, what is the mechanistic interpretation of seeing subunits B, C, D, and E with bound p39 and PSI but not with subunits A and F?

(6) Although quality indicators for the structures such as resolutions were given in Extended Data Fig. 1 and Extended Data Fig. 3, these numbers were barely discussed in the text with respect to their likely impact on the interpretations of the results. Also, the PDB verification report and PDB code for the deposited structures were not provided, which makes evaluation of the quality of these models impossible. It is imperative to provide the information to the reviewers per journal policy. L98: The Extended Data Fig. 1a has an unusual FCS curve for the masked, where the 1.43 line falls onto the flat part of the curve, suggesting that the resolution is a bit over claimed. Another issue is the way that maps were contoured using density thresholds, which needs justification. What features in the map warrant the uses of these numbers? It seems different numbers can be applied in order to show different features. Does it have anything to do with how the map was sharpened or is it related to number of charges per unit volume?

(7) L114-115: The identity of this piece of threading peptide needs more explanation. (1) The authors will need to provide additional evidence to confirm the "substrate" density in the D2 ring is a hairpin of I3, when the resolution of the density is insufficient to assign amino acid sequence. (2) There are several maps produced from different classes, does this substrate density look the same in all these classes. It would be nice to provide a side-by-side views of this substrate density to support this conclusion. (3) Most p97 structures with trapped substrate show the number of residues to be 22 for the substrate. The IRS of I3 starts at residue 25, which seems to suggest that the observed hairpin is too small?

(8) About the structure of p37, it was modeled into discrete pieces of low-resolution densities. L180: Which maps show the extra density at the SHP box binding site? How strong is the extra density? This reviewer doesn't find it very conclusive to have it included in the result section. Perhaps having it in the discussion section is more appropriate. A related question mentioned earlier is how good are the densities for the N-domain? In most previously reported EM structures, N-domains have very poor density. This structure seems no exception. If P97 N-domain is already in low-resolution local regions (~10A), any extra density bulging out of it may be considered noise unless the density is very strong. Additionally, if the conformation of each N domain is not determined accurately, the distance between neighboring N-domains may not be 50Å. Does the linker still long enough to bridge two sites if neighboring N-domains are in different conformations? L185: Need more evidence. For example, flexibility of the linker. Does the sequence of this linker support a random coil with no secondary structure? Fig 3D-G, Fig 5. The authors did not show the density of the p37 N-terminus and SEP domain. With missing density for the 215-residue polypeptide, the authors assumed the SHP motif of p37 occupies the SHP box. With all the missing parts, it seems difficult to conclude the SHP Box binding site actually occupied by P37. Figure 5 doesn't agree with Figure 3. It is very unclear that P37-SHP box on subunit B is from subunit C (figure 3E-G) and SEP from subunit A, especially there is no clear density (reported) for SEP domain.

(9) Maps with SPI binding on subunit C, D, and E show some extra density (or SPI binding on subunit B showing missing density). This is particularly prominent for subunit C in Figure 2a. It would be interesting for the authors to compare these maps and offer a good discussion for these different conformations, rather than focusing on SPI binding to subunit B and making a conclusion.

(10) About the structures of I3, it was assigned into discrete pieces of low-resolution density in the PP1 region, and a detailed structure is not known. L218: Do these observations unique to SPI binding to subunit B? Do the authors observe extra density when SPI binding to other subunits such as subunit D? More importantly, it seems necessary to biochemically confirm the binding site.

Issues with figures.

Extended Data Fig 2H, please indicate the resolution for the map generated by model. Also if possible, please explain why model-map FSC shows better resolution than half-maps fsc?

Fig1B: Identity of the "substrate" needs to be reported in the figure caption.

The distances from Q50, Y143, G54 of p97 to the SDS22 helix should be indicated in Extended Figure 4a.

Minor issues

L85: This paragraph, the last paragraph of the Introduction section, is very vague and insufficient to state the aim of the research. It simply only stated that there is no structural information and poorly understood. It would be nice to have a logical summary of the experiments conducted in this work to be included here.

L85: In the introduction, " .... how protein complexes such as SPI are loaded onto... " Is SPI a complex composed of PP1+SDS22+I3? If so, please define it when appearing for the first time. This is particularly important for uninitiated readers.

L131 "We obtained four individual maps, in which the SPI complex is positioned on the second, third, and fourth highest p97 subunit ...." Shouldn't it be "...second, third, fourth, and fifth ..."?

what was the point of mentioning preliminary data collection under at Bio-Imaging Center different conditions? Was there any data from the preliminary data collection used in the final reconstruction?

Fig ext 3E and 3F. mislabeled subunits

Ext Fig 2B-E, please indicate whether the fsc is masked or unmasked.

For the N- and C-subdomain of the N domain, the standard nomenclature is N-terminal double -barrel and C-terminal four-stranded -barrel.

Response to referees' comments EMBOJ-2022-113110

Structural basis of a hold-and-extract mechanism underlying ubiquitin-independent PP1 complex disassembly by p97

Referee 1

We thank the referee for the positive and constructive comments.

Main points

1) The limited resolution represents a significant challenge in reliably assigning extra densities observed for p37/SPI-bound p97, and the authors seem to overestimate the resolution of their reconstructions. Contrary to the claims in Extended Data Figs. 1c and 3a, the resolution of SDS22, the p97 N-domains, and p37's UBX domain is not in the 7-9 Angstrom range, as that would allow a clear detection of secondary structures. Instead, it seems to lie in the 15 - 20 Angstrom range, which only reveals overall envelopes.
Higher flexibility and potentially alternative conformations appear to limit the resolution in the more peripheral areas of the complex. Have the authors considered re-extracting particles with a focused refinement around the N-terminal domains in order to identify different conformational states with higher resolution?
Without the existing crystal structure of the SHP-box bound to the p97 N-domain, the extra density shown as a small white blob in Fig. 3c would not be sufficient for an assignment to p37. The authors should specify how this extra density was identified, e.g. by creating a difference map using other N-domains in the p97 hexamer.

Limited resolution: The resolution plot in former extended fig 1C (now Expanded View Fig. 2E) is for the p97 domains only, which are mainly well defined with bulky side chain densities visible. This map contains many more particles than the individual classes focussing on the less well-ordered N domains and substrates. We agree that the resolution of the latter domains appears to be worse than the 7-9 Å indicated by the local resolution plot in the current Fig EV4A. This plot was generated by standard software used in the field and is primarily useful for indicating relative resolution of different parts of a structure. Extensive efforts were made to improve the classes, but they do not contain enough data for any further subclassification. However, the density allows clear and unambiguous identification of the N domains and SPI. We do not interpret any side chain detail from these structures.
We have added a comment to the respective figure legend (now Fig. EV4A) to explain that the local resolution plot appears to over-estimate the resolution for N-domains and substrates.

Position of the SHP box: Regarding the SHP box, a difference map was generated between the N domain fit and the density on subunit B and added as a new figure (Appendix Fig. S2). The difference density clearly shows the UBX domain specifically on subunit C and the SHP box density only on subunit B.

2) Interpreting the unassigned densities 1 and 2 in Fig. 4 is even more challenging. It is certainly conceivable that density 1 near the PP1 active site represents a C-terminal part of I3, but the assignment for density 2 is much less convincing. The authors propose that this extra density 2 originates from I3's RVXF motif, ignoring that it is observed several Angstroms away from the apparently empty RVXF-binding site on PP1. In the discussion, it is stated that "we cannot exclude that the disordered N-terminus of SDS22 also contributes to this density", which highlights the need for either further data analyses and classification to reach a higher resolution or the generation of mutants, for instance a N-terminal truncation of SDS22.
In addition, Fig. 2a shows a large, blobby density on SPI bound to the C subunit of p97, which is acknowledged by the authors but not further explained.

We present the RVXF binding as a hypothesis. The observed density does clearly not represent the RVXF motif itself but is adjacent to the established binding site on PP1 and therefore likely represents a sequence stretch close to the RVXF motif. We assume that the RVXF motif is bound at the correct site but cannot be discriminated from the PP1 density.

We edited the text (line 194 and Fig. legend 5) to say that the density is in the vicinity of the RVXF binding site.

Additional density on subunit C complex: The other classes also displayed subsets with a similar additional density, and we were able to sort them into separate classes but we did not have sufficiently good data for the subunit C complex to do this separation. Although there are more particles than some of the other classes, the map is compromised by a strongly preferred orientation. The additional density is too noisy to interpret.
We have added this explanation to Fig. legend 2.

3) The authors propose a hold-and-extract mechanism for the release of I3 from SDS22-PP1 that relies on the assumption that the observed architecture and SPI's interaction with p97 are maintained during active pulling on I3. However, there is no experimental evidence supporting this model, as the reconstructions represent a true initiation complex, in which SPI and p37 interact with hydrolysis-inactive p97 in a state prior to ATPase-driven force application. Whether SDS22-PP1 is held in the same or similar N-domain-bound position during I3 extraction depends on how the strengths of I3 interactions with various regions of PP1 compare to the interaction between the short C-terminal helix of SSD2 and the p97 N-domain cleft, for which there are no experimental data. Although it is possible that an extraction complex looks similar to the presented structure, it is equally conceivable that mechanical tugging on I3 dislodges SDS22-PP1 from its binding site on the N-domain and pulls it against the p97 pore for I3 extraction. The authors should consider this and qualify their claims about an extraction mechanism, including in the title.
Similarly, it is not quite correct that the structures "show p97 ... in the act of translocating a segment of I3", as stated in the first paragraph of the results. Translocation is ATP-hydrolysis dependent, whereas the presented structures originated from SPI binding to a hydrolysis-dead p97 in complex with ADP-BeFx.

To address the referee's concern, we have conducted an additional experiment in which we performed the crosslink (SDS22 to p97 N-domain) in the presence of ATP and compared that to ATPγS, ADP and no nucleotide. The result (now added as Expanded View Fig. EV5B) shows that the SDS22-p97 interaction is stable (and even stronger) in the ATP condition when I3 is threaded through the channel and force is applied to the PP1 complex. We take this as evidence that SDS22 is held in position during PP1 complex disassembly, supporting our model of a hold-and-extract mechanism. We edited the text (line 158) to describe the added data.

4) Based on previous structures of substrate-bound p97 and Cdc48, the central channel accommodates about 20 residues of a substrate polypeptide. If the far N-terminal region of I3 adopts a hairpin conformation, as proposed by the authors, the number of I3 residues inside the channel may be at least 25. This leaves only 15 residues to span the 60 A distance between the RVXF binding site on PP1 and the entrance to the p97 pore, which would be quite a stretch, especially if the authors suggest additional interactions of this I3 region with p97's SEP domain (schematic in Fig.5). Instead, SEP domain interactions may guide I3 toward and into the p97 pore and occur prior to the channel-engaged state observed in the presented structures. The authors should consider these aspects, distances, and number of available I3 residues in more detail. There is also a discrepancy between Fig. 5, which indicates a distance of 60 A between the pore and extra density 2, and the movie, where this distance is given as 50 A.

We thank the referee for pointing out the mistake. The distance is indeed 50 Å which we have corrected in the model (now Fig. 6). We agree that the structure does not inform exactly how the I3 is inserted and reaches the position observed in the structure, especially since we cannot calculate peptide lengths without a precise assignment of the RVXF motif. Based on the previous biochemical evidence demonstrating loop insertion (van den Boom et al., 2021, NSMB) together with the structural information shown here, the most likely scenario is that a loop of I3 (involving the internal recognition site) is guided to the pore and inserted. This can be followed by straightening out the loop of the peptide in the channel with the N-terminus pointing down. In case the N-terminus contains more sequence (or is blocked by experimental circularization as in van den Boom et al., 2021, NSMB), threading occurs as a loop. We agree that the number of available residues is borderline for 50 Å, and have qualified this point in the text (line 202).

5) The authors state in the discussion that the interface of SDS22-PP1 with p97 "is not solely conferred by the adapter" and that "multivalent interactions of adapter, the p97 N-domain and subunits of the SPI complex appear to tightly dock and hold SDS22-PP1 on the p97 N-domain". However, based on the presented structures, the p37 adapter appears to not at all contribute to the interface of SDS22-PP1 with p97 or a tight docking, as there are no p37-SPI interactions observed in these structures. If p37 stabilized SPI, one would expect some density to be resolved. It is possible that the stabilization of the SPI-p97 complex by p37 originates from linking two neighboring N-domains through their interactions with the UBX domain and SHP-box, and it could be helpful to assess whether the two p37-contacted N-domains show any conformational differences compared to the other four N-domains within the hexamer.

Overall, none of the previously proposed interactions between p37 and PP1 or I3 are resolved in these structures, which limits the conclusions that can be drawn about p37's role in SPI processing by p97.

The fact that no interactions of p37 with the PP1 complex were resolved in our structure does not disprove that they exist. Our structure only shows the p97-binding elements of p37 (SHP box and UBX domain). However, there is overwhelming biochemical evidence that p37 is essential for recruiting the PP1 complex to p97 (Weith et al, 2018; Kracht et al., 2020; van den Boom et al., 2021). The previous data highlighted the role of the SEP domain and the SHP-UBX linker of p37 for interaction with the PP1 complex, which are not visible in the structure. We now make clearer in the text that the above-mentioned statements integrate previous biochemical data (line 220).

Minor points:

1) SPI is observed bound to various p97 subunits (B, C, D, and E) that adopt different position within the spiral conformation of the hexamer. The authors present this in a circular arrangement in Fig. 2b, where different states are linked by arrows. This does not seem ideal and may be misinterpreted as SPI "rotating" on top of the p97 hexamer. There may be better ways to show that 1) during initiation SPI can bind p97 subunits irrespective of their position in a spiral arrangement and 2) these states may be equivalent to conformations expected during a subsequent hand-over-hand threading of I37 through the pore.

We agree with the referee. We have removed the arrows and rearranged the figure.

2) The legend for Fig. 4 mentions "side, front, top, and bottom views", but only 3 views are shown.

The legend of what is now Fig. 5 has been corrected.

3) Additional bands detected in the Western blots for SDS22 and p97 in Extended Data Fig. 4b should be explained/labeled.
In the legend for Extended Data Fig. 4c, it needs to say "p97-Q50BpA crosslink sample from (b)" not "(d)".

The band pattern (now main Fig. 4B) is now better labelled with brackets indicating crosslinked p97 multimers, SDS22 and p97. The error in the legend has been corrected.

4) The legend for Extended Data Fig. 5 does not specify how individual components in panels b and c were detected (presumably by Western blotting).

This now refers to Expanded View Fig. 5D and F. We now specify that we performed Western blotting.

<u>Referee 2</u>

We thank the referee for the positive and helpful comments.

1) The identified interaction between SDS22 and p97's N-domain relies on a specific interface, characterized by complimentary protein shapes and electrostatic properties. Given this specific SPI-p97 contact, how do the authors translate their findings to other multiprotein complexes? Could the identified receptor site in p97 serve as general substrate binding patch, e.g. via charge-charge interactions? Or do other clients undergo different yet specific contacts with the N-domain? I was also wondering whether the authors tried to model client complexes with the p97 N-domain, using AF2? Having SPI as positive control, such in silico screening may provide a quick estimate of further candidate complexes.

We agree that these are very interesting points, but given the limited resolution of our substrate densities, we are unfortunately unable to analyse the structure at this level of detail. This would require side chains to be resolved.

2) The structural analysis of the SPI:97 complex is focused on the state where client is bound to subunit B. It seems to be the subunit where the SPI core is best defined but presenting only one of several states. I have questions to the remaining classes: Can the authors comment on the particle distribution of the separate states, which states were most/least populated in EM recordings? Can the authors comment on the unassigned density, attributed to I3 - e.g. is it seen also in other states, even if at lower resolution? Could these different states present a translocation movie, visualizing a gradual discharge of I3 from the SPI complex and/or changes in the density in the central pore (via A-B-C-D-E-F separated states)? It would be great, if the authors could briefly comment on the mechanistic relevance of the separated structures.

The particle numbers in each class are given in Expanded View Fig. 4F.
All the maps show density in the channel. Expanded View Fig. 3B has been added showing the substrate density in the channel for all the states.

It is an appealing idea to order the separated structures into a temporal sequence, but we have no evidence for this.

3) The pBpA crosslinking data look extremely nice. I would suggest moving this figure from the SI to the main part.

Following this suggestion, we have moved the figure to the main part as Fig. 4.

4) Taken the geometry of p37 binding into account - could the SHP-linker-UBX interactions with adjacent p97 N-domains be validated? E.g. shortening the linker by a few residues and preventing its simultaneous interaction by two N-domains? To better illustrate the coupled binding of p37, I would suggest showing the SHP-linker-UBX densities in one figure panel.

We have done linker mutations before and saw effects on substrate binding (Kracht et al., 2020). Analysing linker mutations by cryo-EM would go beyond the time frame of this revision. We now show the distance and orientation of SHP box and UBX domain relative to each other together in one panel in new Appendix Fig. S2.

5) The location of I3 was deduced by "two regions of additional density that are not filled by PP1 or SDS22". Could this point be backed up by functional data? XLMS data or further cryoEM analysis of one of the other p97:SPI states would strengthen this argument.

The binding sites of I3 on PP1 shown in our structure were previously demonstrated by biochemical experiments performed by others (Zhang et al., 2008). The position of the RVXF motif on PP1 is further confirmed by XLMS of the p97-p37-SPI complex in our previous paper (van den Boom et al., 2021). We now explicitly emphasize this in the main text (line 194 and following).

Minor points

(175-190) The authors' make some claims about the positioning of the p37 adaptor, where they conclude p37 is bound in an extended conformation. The reasoning is fitting, but should ideally also comment on the possibility of more than one copy of p37 being bound to p97, rather than both binding sites belonging to the same p37 molecule, and clarify the reasons why they believe it's the same p37 molecule.

We thank the referee for pointing this out. However, previous biochemical evidence showed that the active complex with the SPI substrate contains only one copy of p37 (Kracht et al, 2020). Also, Cooney et al (2019) only observed one UBX domain of the p37 orthologue Shp1. We now include these points in the manuscript text (line 182) to strengthen the argument.

(297) Authors claim that the hold-and-extract mechanism is shown in the present study. However, the role of p37 to hold SPI in place, preventing it to get pulled into the unfoldase, was not fully validated by biochemical data. It may be helpful to state that the „holding" mechanism is rather a hypothesis than a claim. Alternatively, further biochemical evidence is required to discriminate the presenting role of p37 from the alternate hypothesis that the adaptor helps to increase affinity for SPI. In fact, Extended Data Fig. 5 (615-625) suggest a strong effect on the affinity for substrate rather than spurious unfolding due to lack of „holding".

We propose that the "holding" element is conferred by both p37 and the direct interaction between SDS22 and p97-N. Previous biochemical data demonstrated that ablation or mutation of p37 precludes SPI binding to p97 (Weith 2018, Kracht 2020). The role in increasing the affinity and the proposed role in "holding" cannot be separated by our mutations. In response also to reviewer #1, we probed the interaction between SDS22 and p97-N in the presence of ATP (when pulling force is applied) and compared it to ADP and ATPγS (new Expanded View Fig. 5A). The result shows that SDS22 is not dislodged from p97-N during pulling. Nevertheless, we now discuss the "holding" aspect more carefully in the text (line 245).

(555-559) I would encourage the authors to submit the raw micrographs and/or the motion-corrected micrographs to an appropriate repository (EMPIAR). It is unclear whether masks and half-maps were included in the EMDB entries as additional maps - if not, I would

encourage including them. Moreover, please upload the cross-linking MS data to the PRIDE repository.

Half maps are all in the EMDB depositions, the accessions are provided in the "data availability" section. We provide the map with SPI on the B subunit for the referee with this submission. Only the p97 core particle was masked, to exclude the N domains and substrates. No masking was done for the substrate complex which is the new finding reported in this paper. Because of the limited resolution and preferred orientation, the raw images are of limited usefulness and we prefer not to deposit them in EMPIAR. Raw images and a set of 2D classes have been added to Fig. EV2A-D. We have uploaded the requested mass spectrometry data to the PRIDE repository (PXD039939).

(Extended data figure 5, panel b) - the composite image shows Western blots for multiple proteins, some of which have very similar size (SDS22 41 kDa, PP1 37 kDa) and could not have been separated by cutting the membrane in bands of appropriate size. There is no information about whether this data comes from membranes that were stripped and re-treated with a different antibody, or multiple gels were run. Would be reassuring if this information would be included in the methods, or if it was otherwise noted how cross-signalling was avoided.

We thank the reviewer for pointing this out. We have labelled the Figure (now Expanded View Fig. 5D and F) to make clear that the data are from 2 gels/blots.

(Figures) The complementary charges of SDS22 and p97 should be shown, highlighting the two mutated Lys/Arg residues.

We feel this would constitute over-interpretation, since surface charge mapping depends on side chain positions which we do not resolve.

Referee 3

We thank the referee for the positive comments on the novelty of the work.

Major issues

(1) There is a lack of details in describing experimental procedures used in this work, especially in sample preparation. At Line 95 (L95) it says that "The resulting p97-p37-PSI complex was purified by size exclusion chromatography and analyzed by cryo-EM." No details provided. This reviewer requests to see the size exclusion profiles and 1 or 2 EM images to be included in the supplementary data. This will help readers to know more about complex elution volume, stability, and how the complex looks on EM grids. A SDS-PAGE of the peak fraction in the SEC profile containing all components would also be very helpful.

We noted that we made a mistake in the main text. The complex was not purified by SEC for subsequent cryoEM but analyzed directly after assembly. This was correctly described in the

methods section and has now been corrected in the main text (line 103). To address the referee's point, we now show an SEC profile and SDS-PAGE of an example preparation as requested. It demonstrates that the complex is stable with the expected stoichiometry. These data have been added as new Expanded View Fig. 1.

L129: It is also good to know how many classes requested for 2D or 3D classifications? A figure for 2D class averages is needed to show different orientations. Are there any orientation preference among these different 3D classes? It is also not clear whether the structure presented in Figure 1 was produced before the 3D classification or is one of the 4 classes.

For better illustration, 3 raw EM images and a set of 2D class averages were added (new Expanded View Fig. 2A-D). Regarding the number of classes, the analysis was repeated multiple times with different parameters to check reproducibility and optimise the results. 100 2D class averages and between 6 and 10 3D classes were requested.
The orientation plots are provided in Expanded View Fig. 2F (for p97) and Expanded View Fig. 4F (for SPI on p97).
The structure in Fig. 1 was generated before 3D classification into the different substrate complexes, and the substrate domains were masked out. This information has been added to the figure legend.

*(2) Along the same line as in (1), it would be interesting to know why ADP*BeFx was used in this experiment instead of ATPyS reported in their previous experiments (van den Boom et al., NSMB 2021). How relevant is this change in using ADP•BeFx instead of ATPgS with respect to the reaction cycle of the unfolding process should be discussed.*

The transition state analogue ADP-BeFx supports formation of the active staircase conformation with threaded substrate, as shown in many other papers (Twomey (2019), Cooney (2019), Pan (2021). The ATPyS conformation is flat without the substrate inserted.

(3) The paper concludes that all N domains are in the Up conformation without showing experimental data. This reviewer has not seen any p97 structures with good densities for N-domain or in uniform up conformation for wild-type p97. If the authors are so confident about the N-domain conformation, they should show it. Previous works showed the correlation between the conformation of N domain and the cognate D1 nucleotide state. Can the authors make a conclusion on the nucleotide-binding state for each subunit in the D1 and D2 domains? This question becomes important considering the proposed p37 binding between N-domains of neighboring subunits.

The N domains are all shown in their density in Expanded View Fig. 3A (and subunit B, C, and D densities in Fig. 3a), clearly demonstrating that they are in the "up" conformation in all cases. The resolution of our maps is not sufficient for reliable determination of nucleotide states.

(4) It would be interesting to compare current structure with previously published ones. Just by visual inspection, it seems quite different. Can a comparison be made? L105: It was mentioned that subunit F is disordered, bridging subunits A (highest position) and E (lowest

position), referencing Fig.1 and Extended Data Fig. 1. In Figure 1, it is not obvious at all that the F subunit is disordered. In the Extended Figure 1, it does not even mark where the subunit F is. Also, in general how does one distinguish between flexibility and lower occupancy?

We compared our structure with the one by Peter Shen as requested (see new Appendix Fig. S1). The p97 (D1-D2) part of the structures agree with an RMSD with 2.1 A. A comment about this high similarity has been added to the text (line 111).
Subunit F (and all others) are now labelled in what is Ext. Data Fig. 2G in the revised manuscript. Subunit F disorder can be seen by the smaller visible density – eg compare to subunit D in the map top view, Figure 1A. Subunit D shows the N domain and helical hairpin but subunit F does not.
EM classes can't distinguish between flexibility and partial occupancy – both result in weaker density.

(5) L133: please verify that there is no detected class (even at low resolution) with SPI bound to subunit A and F. Or it's just extremely low particle count which can be neglected. L138: The sentence "Subunit B shows ...". This reviewer doubts that this statement is correct. First of all, SPI density looks best when bound to subunit B and D, whereas C and E are a bit poor. However, this can be influenced by the number of particles in each class. B 191,477; C 23,526; D 253,882; E 82,968. So the authors should either focus on the number of particle in each class to draw conclusion that SPI binds to subunit B and D most of the time (occupancy) or normalize the particle size and analyze the resolution and density quality. Furthermore, what is the mechanistic interpretation of seeing subunits B, C, D, and E with bound p39 and PSI but not with subunits A and F?

Fig 2 clearly shows that the substrate density on subunit D is poorly defined and noisy, compared to that on subunit B. Attempts to manually dock the atomic structures into the different maps make this very clear. It was not possible to refine fits of the substrates on the other subunits. This is likely a result of both particle number and the degree of preferred orientation, which was a limiting factor in much of this analysis.
It is not feasible to obtain good classes focussing on either of the subunits at the seam – that is the most mobile part of the structure. Therefore, we can't conclude anything about substrates on A or F, this is simply not observable. Nor can this analysis support a conclusion about the probability of substrate binding to any particular subunit.

(6) Although quality indicators for the structures such as resolutions were given in Extended Data Fig. 1 and Extended Data Fig. 3, these numbers were barely discussed in the text with respect to their likely impact on the interpretations of the results. Also, the PDB verification report and PDB code for the deposited structures were not provided, which makes evaluation of the quality of these models impossible. It is imperative to provide the information to the reviewers per journal policy. L98: The Extended Data Fig. 1a has an unusual FCS curve for the masked, where the 1.43 line falls onto the flat part of the curve, suggesting that the resolution is a bit over claimed. Another issue is the way that maps were contoured using density thresholds, which needs justification. What features in the map warrant the uses of these numbers? It seems different numbers can be applied in order to show different

features. Does it have anything to do with how the map was sharpened or is it related to number of charges per unit volume?

Because the resolution is so variable over the different parts of the structure, the average resolution, which is the main indicator determined by the FSC, does not provide very specific guidance for interpretation. That must be judged on the fit of individual domains into the map. All maps and models reported in this paper have been deposited in the EMDB and PDB, to be released on publication. We make the map with SPI on the B subunit, the PDB code and PDB verification report available for the referees with this submission. Regarding the FSC, the curves were produced by the standard EMDB FSC server. Expanded View Fig. 2E-G refers to the ordered core of the complex, i.e. just the D1 and D2 domains of p97, which do show side chain density and other features consistent with the reported resolution range.
The choice of contour level is indeed chosen to display the features being discussed, and/or to represent the expected volume of the complex. This is normal practice and is used simply for display – the contour level has no bearing on the underlying data. Readers can examine the maps in the EMDB and contour them at any desired level.

(7) L114-115: The identity of this piece of threading peptide needs more explanation. (1) The authors will need to provide additional evidence to confirm the "substrate" density in the D2 ring is a hairpin of I3, when the resolution of the density is insufficient to assign amino acid sequence. (2) There are several maps produced from different classes, does this substrate density look the same in all these classes. It would be nice to provide a side-by-side views of this substrate density to support this conclusion. (3) Most p97 structures with trapped substrate show the number of residues to be 22 for the substrate. The IRS of I3 starts at residue 25, which seems to suggest that the observed hairpin is too small?

We cannot and do not claim that I3 is in a hairpin conformation but raise the possibility based on previous biochemical data. It is possible that the IRS is recognized and inserted into the pore as a loop, but that the peptide then straightens within the channel so that the N-terminus points towards D2. When additional sequence is added to the N-terminus experimentally (van den Boom et al., 2021), threading is mediated by a loop. We now show in new Ext Data Fig. 3B that peptide density is present irrespective of whether SPI is located on subunits B, C, D, or E.

(8) About the structure of p37, it was modeled into discrete pieces of low-resolution densities. L180: Which maps show the extra density at the SHP box binding site? How strong is the extra density? This reviewer doesn't find it very conclusive to have it included in the result section. Perhaps having it in the discussion section is more appropriate. A related question mentioned earlier is how good are the densities for the N-domain? In most previously reported EM structures, N-domains have very poor density. This structure seems no exception. If P97 N-domain is already in low-resolution local regions (~10A), any extra density bulging out of it may be considered noise unless the density is very strong. Additionally, if the conformation of each N domain is not determined accurately, the distance between neighboring N-domains may not be 50Å. Does the linker still long enough to bridge two sites if neighboring N-domains are in different conformations? L185: Need

more evidence. For example, flexibility of the linker. Does the sequence of this linker support a random coil with no secondary structure? Fig 3D-G, Fig 5. The authors did not show the density of the p37 N-terminus and SEP domain. With missing density for the 215-residue polypeptide, the authors assumed the SHP motif of p37 occupies the SHP box. With all the missing parts, it seems difficult to conclude the SHP Box binding site actually occupied by P37. Figure 5 doesn't agree with Figure 3. It is very unclear that P37-SHP box on subunit B is from subunit C (figure 3E-G) and SEP from subunit A, especially there is no clear density (reported) for SEP domain.

We clearly see the extra density on the SHP box binding site in the map with SPI bound on the B subunit. We added a difference map between the density generated by the N domain fit and the density on subunit B in Appendix Fig. S2 to improve the presentation of the extra density. The resolutions of the other maps do not allow a clear assignment whether the SHP box binding site is occupied.
The N domains are clearly shown (now Expanded View Fig. 3A), all in the up position. The densities unambiguously define the domain shapes and orientations, even in the case of subunit F which is the most disordered.
We corrected the model figure to make clearer that the SEP is not on subunit A.
The SHP-Ubx linker is predicted to be unstructured by alpha fold 2 and could easily bridge the observed positions of adjacent N-domains.

(9) Maps with SPI binding on subunit C, D, and E show some extra density (or SPI binding on subunit B showing missing density). This is particularly prominent for subunit C in Figure 2a. It would be interesting for the authors to compare these maps and offer a good discussion for these different conformations, rather than focusing on SPI binding to subunit B and making a conclusion.

The other maps are too noisy to do a valid comparison, as far as we can tell the SPI binds to all of them in the same way. As mentioned in the reply to reviewer 1, point 2 (and noted in the revised figure legend), the subunit C map is particularly noisy and we were not able to separately classify a complex without the extra, unidentified density above the channel.

(10) About the structures of I3, it was assigned into discrete pieces of low-resolution density in the PP1 region, and a detailed structure is not known. L218: Do these observations unique to SPI binding to subunit B? Do the authors observe extra density when SPI binding to other subunits such as subunit D? More importantly, it seems necessary to biochemically confirm the binding site.

The lower quality of the substrate density on the other subunits does not allow us to address the point about potential difference. The binding sites of I3 on PP1 have been previously characterized biochemically through mapping and crosslinking (Zhang et al., 2009; van den Boom, 2021). The complex is very stable as now also shown by SEC (new Expanded View Fig. 1)

Issues with figures.

Extended Data Fig 2H, please indicate the resolution for the map generated by model. Also if possible, please explain why model-map FSC shows better resolution than half-maps fsc?

We apologise, but we do not understand what is meant by “resolution for the map generated by model".
The FSCs for all the substrate complexes seem over optimistic, perhaps due to the amount of data and preferred orientation. The FSCs were determined by the EMDB FSC server.

Fig1B: Identity of the "substrate" needs to be reported in the figure caption.

Edited.

The distances from Q50, Y143, G54 of p97 to the SDS22 helix should be indicated in Extended Figure 4a.

The distance to Q50 was already given in what is now main Fig. 4D. We added the other distances in the legend of Expanded View Fig. 5C because otherwise the figure becomes too crowded.

Minor issues

L85: This paragraph, the last paragraph of the Introduction section, is very vague and insufficient to state the aim of the research. It simply only stated that there is no structural information and poorly understood. It would be nice to have a logical summary of the experiments conducted in this work to be included here.

The following paragraph has been added:

"Here we have analyzed assemblies of p97 with its p37 adapter and the SPI substrate complex in the presence of ADP-BeFx by cryo-EM, yielding a set of structures of p97-p37 during the disassembly of the SPI substrate. Unexpected findings provide new insights into the spatial arrangement of a ubiquitin-independent substrate and the adapter protein on p97 that facilitates complex disassembly."

L85: In the introduction, " .... how protein complexes such as SPI are loaded onto... " Is SPI a complex composed of PP1+SDS22+I3? If so, please define it when appearing for the first time. This is particularly important for uninitiated readers.

We have now added the definition.

L131 "We obtained four individual maps, in which the SPI complex is positioned on the second, third, and fourth highest p97 subunit ...." Shouldn't it be "...second, third, fourth, and fifth ..."?

Corrected

what was the point of mentioning preliminary data collection under at Bio-Imaging Center different conditions? Was there any data from the preliminary data collection used in the final reconstruction?

We are required to acknowledge any use of the National facility, even though the data were not used in the final reconstruction.

Fig ext 3E and 3F. mislabeled subunits

Figure corrected

Ext Fig 2B-E, please indicate whether the fsc is masked or unmasked.

The FSCs in what is now Expanded View Fig. 4B-E were calculated without masking.

For the N- and C-subdomain of the N domain, the standard nomenclature is N-terminal double Ψ-barrel and C-terminal four-stranded β-barrel.

We have now implemented this nomenclature.

1st Revision - Editorial Decision 19th Apr 2023

Prof. Hemmo Meyer
Universitat Duisburg-Essen
Faculty of Biology
Institute of Molecular Biology 1
Campus Essen
Essen 45117
Germany

19th Apr 2023

Re: EMBOJ-2022-113110R
Structural basis of a hold-and-extract mechanism underlying ubiquitin-independent PP1 complex disassembly by p97

Dear Hemmo and Helen,

Thank you again for submitting your revised manuscript for our consideration. I apologize for the delay in its re-evaluation - one of the structural experts (referee 2) was initially unavailable for an extended period, but since especially the other structural reviewer (referee 3) turned out to retain substantive reservations at this point, I decided to re-contact referee 2 after all and consult with them regarding the remaining concerns of referees 1 and 3; please find all this summarized in the below-copied comments.
Since referee 2 brought good reasons for why the points raised by referee 3 should not strongly affect the validity of the results and the strength of the conclusions being drawn, I concluded that we will not require further experimental revisions in this case. I would however invite you to carefully respond to referee 3's report and, if necessary, also include additional clarifications or disclaimers in the text.
Furthermore, referee 1 found that certain statements that were not sufficiently justified by the present dataset should still be toned down; and given that referee 2 also agreed with this view, I would kindly ask you to make the requested adjustments in a final version of the text, ideally with "track changes" activated to facilitate my final checking.

Everything else seems to be in order now, and once we will have received your final minor revision, we should be ready to swiftly proceed with formal acceptance and publication of the manuscript!

Sorry again for the delay and kind regards,

Hartmut

Hartmut Vodermaier, PhD
Senior Editor, The EMBO Journal
h.vodermaier@embojournal.org

Use the link below to submit your revision:

Link Not Available

Referee #1:

The authors thoroughly addressed the reviewers' criticism and strengthened the paper through additional experiments, analyses, and editing. The paper is therefore now overall suited for publication in EMBO.
Nevertheless, some of the claims should be further toned down. For instance, the header of the first results section still states "Human p97 in the act of translocating a specific substrate protein", which is not correct. The structures show the p97-SPI complex in states that were reached by binding and diffusion in the absence of any ATP hydrolysis, and may therefore primarily represent an initiation complex. Although some of the features may be similar to an actively translocating p97, in part thanks to using ADP-BeFx that induces a spiral staircase arrangement of ATPase subunits, there could also be significant differences, especially regarding the positions of SPI components on top of p97. It is certainly not the case that the authors trapped and visualized p97 in the act of translocating a substrate, and it should therefore also not be stated. This was not addressed in the revision.

For the same reasons, one has to be conservative with claims about a hold-and-extract mechanism and that SDS22's position on p97 remains unchanged during the mechanical force application on the PP1 complex. The newly added crosslinking experiments between p97 Q50Bpa in the presence of ATP indicate that SDS22 occupies an N-domain bound position for a considerable amount of time during the entire PP1-I3 processing (including initial binding), but whether SPI indeed adopts a conformation during mechanical pulling that is similar to the one observed here for initiation remains unclear. For instance, it is conceivable that SDS22 gets pulled against the p97 pore, while still maintaining some contacts to an N-domain that allows crosslinking to Bpa50.

Referee #2:

The authors have mostly addressed the comments of the referees and improved their manuscript. However, there is some disagreement between Referee #1 and #3 on the quality of the structural work and so I give my opinion below.

To me the local resolution looks approximately correct. Secondary structure elements are visible in the N domains and PSI complex, and based on previously known complex structures, these can be docked with high confidence. While the SHP box is poorly resolved, the structure of a homologous interaction is already known, and there is clearly extra density exactly where the peptide is known to bind. The authors are not interpreting high resolution features in their maps, such as side chain interactions, and so a resolution sufficient for docking is sufficient to support their conclusions. To emphasise this point, the authors have converted their models to poly-alanine chains. Similarly, they do not make claims about the translocation mechanism, which has already previously been investigated by others at high resolution, so their modelling of the translocated peptide is not important for their conclusions. Discussion about other lower resolution unmodelled density is clearly framed as speculative in the manuscript.

However, I would agree with Referee #1's comments regarding the translocation state, and once addressed, the paper would be suited for publication in EMBO.

Referee #3:

This is a structural paper describing authors' effort to obtain structural basis at high resolution by cryo-EM for the binding and subsequent activation of PP1 by disassembling a protein complex called PSI by AAA protein p97 unfoldase. Because this is a structural paper, the work must conform to the standard practice in the field. Unfortunately, after carefully examining the EM density file and the accompanying pdb file, as well as the PDB validation report, which were made available with the revision, this reviewer has to say that the work is far from satisfactory. This view seems shared Looking at other reviewers comments. The EM density provided does not support the resolution claimed in the paper. The modeling of the EM density is terrifying with only poly alanine models for ALL protein chains. Even with all polyA chains, the clash scores are high pages long. The density to model correlation as reflected by the Q score is very poor, especially for the X and Y chains. Astonishingly, for the N-terminal part of the inhibitor (I chain) running through the p97 pore, the chain was built backward. The interpretation of the map is also problematic. First, there was no continuous density for the N-terminal I chain in the p97 center pore. The fitting of the SHP box peptide is by imagination even at a contour level of 2 sigma (the recommended 0.0026 level by the pdb validation report). The EM density for the N-domains for A and F chains at 1, 2, or higher sigma contour level does not exist in the map provided, calling into question the claim that ALL N-domains are in the up conformation.

5 May 2023
Structural basis of ubiquitin-independent PP1 complex disassembly by p97
EMBOJ-2022-113110R1

Response to reviewers

Reviewers 1 and 2
We thank the reviewers and have changed the title and the claims about the translocation state in accordance with reviewer 1's comments.

Reviewer 3

This reviewer finds it "terrifying" that the models are provided as polyA chains. We deliberately deposited backbone structures to avoid over interpretation of our limited resolution density. As pointed out by reviewer 2, the density was sufficient to confidently guide the docking of known domains, and our interpretation does not depend on side chain features. The clashes listed in the PDB validation report are all less than 1 Å, beyond the scope of our analysis.

The N-domain densities for chains A and F are weak, but we do not agree that they "do not exist in the map" at 1 sigma. The attached snapshot (Fig R1) shows the N domains in magenta with the density contoured at the recommended level of 0.0026. Even for the A domain which is on the most mobile subunit, there is partial density at that contour level. Regarding the SHP box, we did not use the density to build the peptide structure but rather to demonstrate that it accommodates the peptide interaction previously determined for that binding site. The peptide is shown in dark blue in the zoomed in view, at the 0.0026 contour level (Fig R2).

The reviewer is correct that the segment of I3 in the pore was built backwards. We apologize for this error and thank the reviewer for spotting it. It has been corrected. The updated model and preliminary validation report are attached.

Fig R1. N domains in magenta, map contour 0.0026.

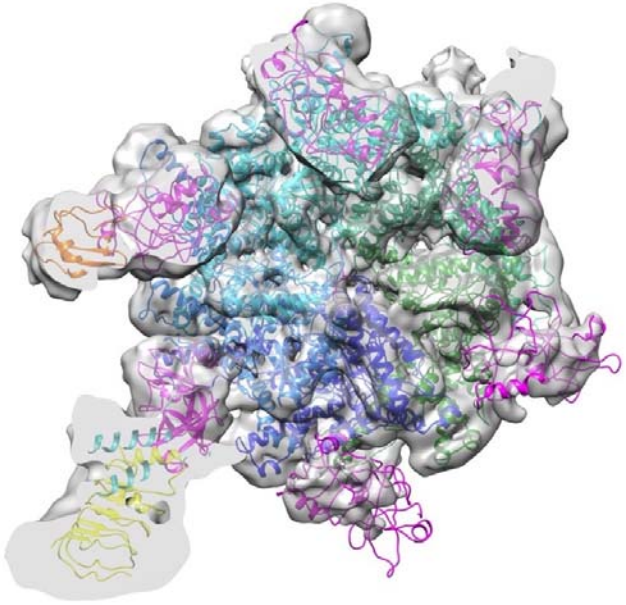

Fig R2. SHP peptide in dark blue, map contour 0.0026.

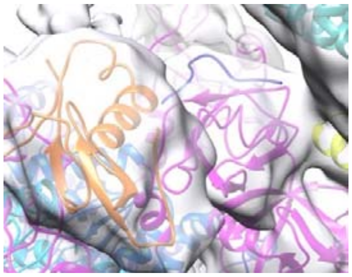

Prof. Hemmo Meyer
Universitat Duisburg-Essen
Faculty of Biology
Institute of Molecular Biology 1
Campus Essen
Essen 45117
Germany

22nd May 2023

Re: EMBOJ-2022-113110R1
Structural basis of ubiquitin-independent PP1 complex disassembly by p97

Dear Helen and Hemmo,

Thank you for submitting your final revised manuscript for our consideration. I am pleased to inform you that we have now accepted it for publication in The EMBO Journal.

Your article will be processed for publication in The EMBO Journal by EMBO Press and Wiley, who will contact you with further information regarding production/publication procedures and license requirements. You will also be provided with page proofs after copy-editing and typesetting of main manuscript and expanded view figure files.

Should you be planning a Press Release on your article, please get in contact with embojournal@wiley.com as early as possible, in order to coordinate publication and release dates.

Thank you again for this contribution to The EMBO Journal and congratulations on a successful publication! Please consider us again in the future for your most exciting work.

With kind regards,

Hartmut

Hartmut Vodermaier, PhD
Senior Editor, The EMBO Journal
h.vodermaier@embojournal.org

Please note that it is EMBO Journal policy for the transcript of the editorial process (containing referee reports and your response letter) to be published as an online supplement to each paper. If you do NOT want this, you will need to inform the Editorial Office via email immediately. More information is available here:
https://www.embopress.org/page/journal/14602075/authorguide#transparentprocess

EMBO Press Author Checklist

Corresponding Author Name: Helen R. Saibil	
Journal Submitted to: EMBO Journal	
Manuscript Number: EMBOJ-2022-113110R	

USEFUL LINKS FOR COMPLETING THIS FORM
The EMBO Journal - Author Guidelines
EMBO Reports - Author Guidelines
Molecular Systems Biology - Author Guidelines
EMBO Molecular Medicine - Author Guidelines

Reporting Checklist for Life Science Articles (updated January
This checklist is adapted from Materials Design Analysis Reporting (MDAR) Checklist for Authors. MDAR establishes a minimum set of requirements in transparent reporting in the life sciences (see Statement of Task: 10.31222/osf.io/9sm4x). Please follow the journal's guidelines in preparing your
Please note that a copy of this checklist will be published alongside your article.

Abridged guidelines for figures
1. Data
The data shown in figures should satisfy the following conditions:
- ➞ the data were obtained and processed according to the field's best practice and are presented to reflect the results of the experiments in an accurate and unbiased manner.
- ➞ ideally, figure panels should include only measurements that are directly comparable to each other and obtained with the same assay.
- ➞ plots include clearly labeled error bars for independent experiments and sample sizes. Unless justified, error bars should not be shown for technical
- ➞ if n<5, the individual data points from each experiment should be plotted. Any statistical test employed should be justified.
- ➞ Source Data should be included to report the data underlying figures according to the guidelines set out in the authorship guidelines on Data

2. Captions
Each figure caption should contain the following information, for each panel where they are relevant:
- ➞ a specification of the experimental system investigated (eg cell line, species name).
- ➞ the assay(s) and method(s) used to carry out the reported observations and measurements.
- ➞ an explicit mention of the biological and chemical entity(ies) that are being measured.
- ➞ an explicit mention of the biological and chemical entity(ies) that are altered/varied/perturbed in a controlled manner.
- ➞ the exact sample size (n) for each experimental group/condition, given as a number, not a range;
- ➞ a description of the sample collection allowing the reader to understand whether the samples represent technical or biological replicates (including how many animals, litters, cultures, etc.).
- ➞ a statement of how many times the experiment shown was independently replicated in the laboratory.
- ➞ definitions of statistical methods and measures:
  - common tests, such as t-test (please specify whether paired vs. unpaired), simple χ2 tests, Wilcoxon and Mann-Whitney tests, can be unambiguously identified by name only, but more complex techniques should be described in the methods section;
  - are tests one-sided or two-sided?
  - are there adjustments for multiple comparisons?
  - exact statistical test results, e.g., P values = x but not P values < x;
  - definition of 'center values' as median or average;
  - definition of error bars as s.d. or s.e.m.

Please complete ALL of the questions below.
Select "Not Applicable" only when the requested information is not relevant for your study.

Materials

Newly Created Materials	Information included in the manuscript?	In which section is the information available? (Reagents and Tools Table, Materials and Methods, Figures, Data Availability Section)
New materials and reagents need to be available; do any restrictions apply?	Yes	Materials and Methods and Data Abailability Section

Antibodies	Information included in the manuscript?	In which section is the information available? (Reagents and Tools Table, Materials and Methods, Figures, Data Availability Section)
For **antibodies** provide the following information:<br>- Commercial antibodies: RRID (if possible) or supplier name, catalogue number and or/clone number<br>- Non-commercial: RRID or citation	Yes	Materials and Methods

DNA and RNA sequences	Information included in the manuscript?	In which section is the information available? (Reagents and Tools Table, Materials and Methods, Figures, Data Availability Section)
**Short novel DNA or RNA including primers, probes**: provide the sequences.	Not Applicable	no novel sequences

Cell materials	Information included in the manuscript?	In which section is the information available? (Reagents and Tools Table, Materials and Methods, Figures, Data Availability Section)
**Cell lines:** Provide species information, strain. Provide accession number in repository **OR** supplier name, catalog number, clone number, and/**OR** RRID.	Not Applicable	
**Primary cultures:** Provide species, strain, sex of origin, genetic modification status.	Not Applicable	
Report if the cell lines were recently **authenticated** (e.g., by STR profiling) and tested for mycoplasma contamination.	Not Applicable	

Experimental animals	Information included in the manuscript?	In which section is the information available? (Reagents and Tools Table, Materials and Methods, Figures, Data Availability Section)
**Laboratory animals or Model organisms:** Provide species, strain, sex, age, genetic modification status. Provide accession number in repository **OR** supplier name, catalog number, clone number, **OR** RRID.	Not Applicable	
**Animal observed in or captured from the field:** Provide species, sex, and age where possible.	Not Applicable	
Please detail **housing and husbandry conditions**.	Not Applicable	

Plants and microbes	Information included in the manuscript?	In which section is the information available? (Reagents and Tools Table, Materials and Methods, Figures, Data Availability Section)
**Plants:** provide species and strain, ecotype and cultivar where relevant, unique accession number if available, and source (including location for collected wild specimens).	Not Applicable	
**Microbes:** provide species and strain, unique accession number if available, and source.	Not Applicable	

Human research participants	Information included in the manuscript?	In which section is the information available? (Reagents and Tools Table, Materials and Methods, Figures, Data Availability Section)
If collected and within the bounds of privacy constraints report on age, sex and gender or ethnicity for all study participants.	Not Applicable	

Core facilities	Information included in the manuscript?	In which section is the information available? (Reagents and Tools Table, Materials and Methods, Figures, Data Availability Section)
If your work benefited from core facilities, was their service mentioned in the acknowledgments section?	Yes	Acknowledgements

Design

Study protocol	Information included in the manuscript?	In which section is the information available? (Reagents and Tools Table, Materials and Methods, Figures, Data Availability Section)
If study protocol has been **pre-registered, provide DOI in the manuscript**. For clinical trials, provide the trial registration number **OR** cite DOI.	Not Applicable	
Report the **clinical trial registration number** (at ClinicalTrials.gov or equivalent), where applicable.	Not Applicable	

Laboratory protocol	Information included in the manuscript?	In which section is the information available? (Reagents and Tools Table, Materials and Methods, Figures, Data Availability Section)
Provide DOI OR other citation details if **external detailed step-by-step protocols** are available.	Not Applicable	

Experimental study design and statistics	Information included in the manuscript?	In which section is the information available? (Reagents and Tools Table, Materials and Methods, Figures, Data Availability Section)
Include a statement about **sample size** estimate even if no statistical methods were used.	Yes	Materials and Methods
Were any steps taken to minimize the effects of subjective bias when allocating animals/samples to treatment (e.g. **randomization procedure**)? If yes, have they been described?	Not Applicable	
Include a statement about **blinding** even if no blinding was done.	Yes	Materials and Methods
Describe **inclusion/exclusion criteria** if samples or animals were excluded from the analysis. Were the criteria pre-established?<br><br>If sample or data points were omitted from analysis, report if this was due to attrition or intentional exclusion and provide justification.	Not Applicable	
For every figure, are **statistical tests** justified as appropriate? Do the data meet the assumptions of the tests (e.g., normal distribution)? Describe any methods used to assess it. Is there an estimate of variation within each group of data? Is the variance similar between the groups that are being statistically compared?	Not Applicable	

Sample definition and in-laboratory replication	Information included in the manuscript?	In which section is the information available? (Reagents and Tools Table, Materials and Methods, Figures, Data Availability Section)
In the figure legends: state number of times the experiment was **replicated** in laboratory.	Not Applicable	
In the figure legends: define whether data describe **technical or biological replicates**.	Not Applicable	

Ethics

Ethics	Information included in the manuscript?	In which section is the information available? (Reagents and Tools Table, Materials and Methods, Figures, Data Availability Section)
Studies involving **human participants**: State details of **authority granting ethics approval** (IRB or equivalent committee(s), provide reference number for approval.	Not Applicable	
Studies involving **human participants**: Include a statement confirming that **informed consent** was obtained from all subjects and that the experiments conformed to the principles set out in the WMA Declaration of Helsinki and the Department of Health and Human Services Belmont Report.	Not Applicable	
Studies involving **human participants:** For publication of **patient photos**, include a statement confirming that consent to publish was obtained.	Not Applicable	
Studies involving experimental **animals**: State details of **authority granting ethics approval** (IRB or equivalent committee(s), provide reference number for approval. Include a statement of compliance with ethical regulations.	Not Applicable	
Studies involving **specimen and field samples:** State if relevant **permits** obtained, provide details of authority approving study; if none were required, explain why.	Not Applicable	

Dual Use Research of Concern (DURC)	Information included in the manuscript?	In which section is the information available? (Reagents and Tools Table, Materials and Methods, Figures, Data Availability Section)
Could your study fall under dual use research restrictions? Please check biosecurity documents and list of **select agents and toxins** (CDC): https://www.selectagents.gov/sat/list.htm	Not Applicable	
If you used a select agent, is the security level of the lab appropriate and reported in the manuscript?	Not Applicable	
If a study is subject to dual use research of concern regulations, is the name of the **authority granting approval and reference number** for the regulatory approval provided in the manuscript?	Not Applicable	

Reporting

The MDAR framework recommends adoption of discipline-specific guidelines, established and endorsed through community initiatives. Journals have their own policy about requiring specific guidelines and recommendations to complement MDAR.

Adherence to community standards	Information included in the manuscript?	In which section is the information available? (Reagents and Tools Table, Materials and Methods, Figures, Data Availability Section)
State if relevant guidelines or checklists (e.g., **ICMJE, MIBBI, ARRIVE, PRISMA**) have been followed or provided.	Not Applicable	
For **tumor marker prognostic studies,** we recommend that you follow the **REMARK** reporting guidelines (see link list at top right). See author guidelines, under 'Reporting Guidelines'. Please confirm you have followed these guidelines.	Not Applicable	
For **phase II and III randomized controlled trials**, please refer to the **CONSORT** flow diagram (see link list at top right) and submit the CONSORT checklist (see link list at top right) with your submission. See author guidelines, under 'Reporting Guidelines'. Please confirm you have submitted this list.	Not Applicable	

Data Availability

Data availability	Information included in the manuscript?	In which section is the information available? (Reagents and Tools Table, Materials and Methods, Figures, Data Availability Section)
Have **primary datasets** been deposited according to the journal's guidelines (see 'Data Deposition' section) and the respective accession numbers provided in the Data Availability Section?	Yes	Data Availability Section
Were **human clinical and genomic datasets** deposited in a public access-controlled repository in accordance to ethical obligations to the patients and to the applicable consent agreement?	Not Applicable	
Are **computational models** that are central and integral to a study available without restrictions in a machine-readable form? Were the relevant accession numbers or links provided?	Not Applicable	
If publicly available data were reused, provide the respective **data citations in the reference list**.	Yes	Reference List
